# Alkali-modified heterogeneous Pd-catalyzed synthesis of acids, amides and esters from aryl halides using formic acid as the CO precursor†

**DOI:** 10.1039/d1ra05177f

**Published:** 2021-08-05

**Authors:** Charles O. Oseghale, Oluwatayo Racheal Onisuru, Dele Peter Fapojuwo, Batsile M. Mogudi, Pule Petrus Molokoane, Nomathamsanqa Prudence Maqunga, Reinout Meijboom

**Affiliations:** Research Center for Synthesis and Catalysis, Department of Chemical Sciences, University of Johannesburg PO Box 524, Auckland Park 2006 Johannesburg South Africa rmeijboom@uj.ac.za +27 11 559 2819 +27 72 894 0293

## Abstract

To establish an environmentally friendly green chemical process, we minimized and resolved a significant proportion of waste and hazards associated with conventional organic acids and molecular gases, such as carbon monoxide (CO). Herein, we report a facile and milder reaction procedure, using low temperatures/pressures and shorter reaction time for the carboxyl- and carbonylation of diverse arrays of aryl halides over a newly developed cationic Lewis-acid promoted Pd/Co_3_O_4_ catalyst. Furthermore, the reaction proceeded in the absence of acid co-catalysts, and anhydrides for CO release. Catalyst reusability was achieved *via* scalable, safer, and practical reactions that provided moderate to high yields, paving the way for developing a novel environmentally benign method for synthesizing carboxylic acids, amides, and esters.

## Abstract 2

To establish an environmentally friendly green chemical process, we minimized and resolved a significant proportion of waste and hazards associated with conventional organic acids and molecular gases, such as carbon monoxide (CO).

## Graphical abstract

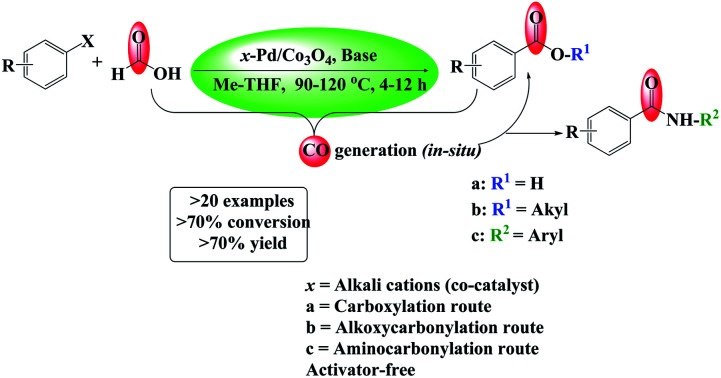

## Introduction

Carbonylation reactions involving transition metals have proven to be the most convenient and versatile method for synthesizing carbonyl-containing compounds such as anhydrides, esters, amides, aldehydes, alcohols, ketones, and acids,^1–6^ which have found applications in a variety of biologically active compounds, pharmaceuticals, and natural products. As a result, the carbonylation process has become an indispensable building block and a highly effective technique for synthesizing fine and bulk chemicals in academia and industry. However, carbonylation reactions have not been extensively used in a laboratory setting for chemical synthesis. This is due to the handling of extremely flammable and toxic CO gas, which works under higher pressure and requires sophisticated stainless-steel autoclave reactors. To address this issue on a laboratory scale, much emphasis has been directed towards the use of CO surrogates.^7,8–10^ Numerous surrogates, including aldehydes,^11^ formic acids,^7,12–15^ and formates,^16,17^ have been used in carbonylation processes catalyzed by transition metals. Among these CO substitutes, formic acid (HCOOH) is the most practical surrogate since it can be readily obtained *via* CO_2_ hydrogenation^18,19^ and bio-waste fermentation.^20,21^ However, the ambiguous breakdown route of HCOOH to H_2_ and CO_2_ or CO and H_2_O remains a common issue with the effective use of formic acid as a surrogate.

The decomposition of formic acid to H_2_ and CO_2_ has been successfully achieved using a variety of homogeneous catalysts,^22,23^ whereas the decomposition of formic acid to CO and H_2_O is currently being investigated owing to the difficulty encountered in its transformation at lower temperatures. Although formic acid dehydration in the presence of strong mineral acids, such as sulfuric acid, may produce CO gas preferentially. The acid is thought to act as an activator for the CO release but has compatibility issues when applied to other carbonylation reactions owing to the process's harsh reaction conditions. Similarly, acetic anhydride,^19,24,25^ triethylamine,^7^ and others have been reportedly employed as activators for selective CO release. However, acetic anhydride requires a stoichiometric amount to promote CO production, which generates waste and complicates the work. As a result, the quest for more environmentally friendly methods to perform these reactions *in situ* in solution persists.

Developing a stable and reusable heterogeneous catalyst to perform carbonylation reactions under these conditions is highly desirable. Although homogeneous catalysis remains vital in academia and chemical industrial processes, it is estimated to account for more than 20% of catalytic reactions in the chemical industry today.^26–28^ Carbonylation reactions catalyzed by transition metals have developed into a critical tool in organic synthesis. These reactions have been carried out using a variety of catalytically active metals. The palladium metal-catalyzed addition of CO to alkynes and alkenes in the presence of a suitable nucleophile has garnered significant interest in recent years for its potential use in synthesizing a wide variety of essential chemical products. Palladium-supported phosphine functionalized porous polymers and various palladium–phosphine complexes have been extensively described.^29–31^ However, these synthetic protocols often require the use of non-recyclable palladium precursors, a longer reaction time, and phosphine ligands, as well as harsh reaction conditions. In addition, these protocols sometimes intricate the carbonylation of the unprotected nitrogen in the phosphine ligands. Moisture-sensitive phosphine ligands and non-recyclable palladium precursors pose significant challenges in terms of catalyst recovery, structural flexibility, application, accessibility, leaching, and reusability.^32,33^ This necessitates the development of highly reusable, economically viable, and environmentally benign carbonylation catalysts. Recently, researchers have developed a method for immobilizing clusters or metallic compounds (palladium complexes) on solid supports such as organic polymers,^34,35^ Fe_3_O_4_^,36^ SBA-15,^37,38^ MOF-5,^39^ ZIF-8 (ref. 40) that is sustainable, green, easy, and cost-effective. The heterogenization of homogeneous catalysts has been extensively researched recently to address sustainability concerns about recyclability, besides designing new ligands to enrich the homogeneous catalysts.

As part of our ongoing work on heterogeneous catalyzed carbonylation reactions, we became motivated to fill the gap by designing a new palladium heterogeneous catalyst for these reactions. The catalytic system utilizes the synergistic combination of alkali metals as ion-promoters on the palladium supported on Co_3_O_4_. The spinel mesoporous Co_3_O_4_ is versatile and constitutes an interesting class of nanomaterials for noble metal support in vast catalytic reactions due to the intrinsic catalytic activity they exhibit.^41–44^ Notably, incorporating dopants such as alkali metals into catalysts has developed into an interesting and active field in catalysis. We recently reported that alkali metals might induce a phenomenon and improve the overall catalyst activity.^2,41,45^ Various studies have documented the increased catalytic activity of these alkali-promoted catalysts due to their electronic and structural effects on these nanomaterials.^46–48^ It was discovered in this study that doping with alkali metals stabilized and induced the active dispersed palladium species on reducible Co_3_O_4_, thus minimizing active site leaching, acting as a potential “in-built” cationic Lewis acid co-catalyst, and improving the catalyst's binding properties.

**Scheme 1:**
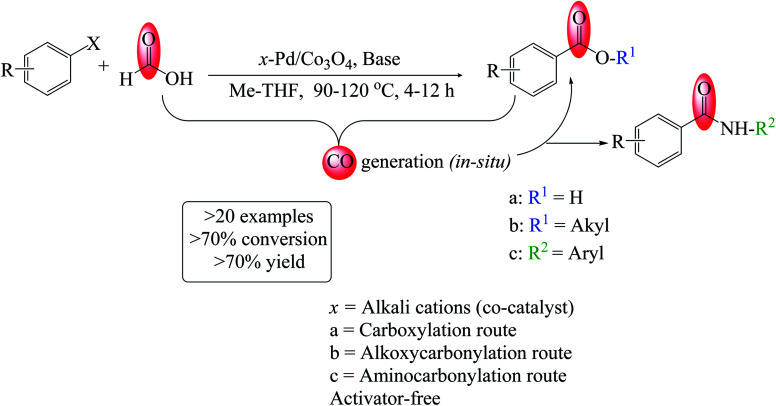
Alkali-cation promoted Pd/Co_3_O_4_-catalyzed carboxy- and carbonylation of aryl halides.

Furthermore, between the generated CO molecules, the newly designed catalyst, and the substrates or products that may limit the substrate's scope, reaction design must account for the undesirable chemistry associated with these reactions. As a result, performing carbonylation reactions on a laboratory scale with various substrates has thus far remained elusive. The study reports a highly reusable catalyst and an operationally simple strategy for the stoichiometric carbonylation reaction under milder conditions (Scheme 1). Notably, this new system exhibited superior activity for the reactions in the absence of acid co-catalysts and anhydrides (activators). The critical success may be attributed to CO generated *in situ* in the solution containing the ion-promoted Pd-catalyst. We anticipated that this catalytic system would improve the rates of carboxy- and carbonylation reactions by utilizing formic acid as a CO precursor. Additionally, we demonstrated that this green method might be used to effectively synthesize many biologically active amide compounds. Notably, this facile protocol serves as a critical tool for drug development programs and discovery.

## Experimental section

### Chemicals

Apart from the calcium (98%) and potassium (>99%) nitrate salts purchased from Associated Chemical Enterprises, all other chemicals used in this work were of analytical grade and purchased from Sigma-Aldrich. The reactions were performed using an autoclave and a drilled aluminum block with securely capped 20 mL dram-vials at different times. In addition, high purity solvents from commercial suppliers were used without the need for further purification.

### Synthesis of the catalysts

The deposition–precipitation synthetic technique was used to synthesize undoped Pd/Co_3_O_4_ and alkali metal (x = Cs, Na, Li, Ca and K) doped Pd/Co_3_O_4_ catalysts with the same alkali metal/Pd mole ratio (2% mol/mol).^49^ Typically, 1 g of the as-prepared Co_3_O_4_ (ref. 2) was added to an aqueous Pd(OAc)_2_ solution and stirred at room temperature (RT) for 2 h. Urea : Pd salt at a (2 : 1) molar ratio was added to the reaction mixture with heating and stirring for 1 h at 80 °C. After cooling to room temperature, freshly prepared NaBH_4_ was added to the reaction mixture at a 10 : 1 molar excess and agitated for a further 4 h at RT. The alkali metal was then added to the mixture and stirred for 1 h. The precipitate obtained was washed with 100 mL of excess distilled water, dried, and calcined for 3 h at 350 °C. Prior to conducting the activity tests, the catalysts were reduced in H_2_ for 20 min at 350 °C and labeled as Co_3_O_4_ (X1) and 3% Pd/Co_3_O_4_ (X2). The doped equivalents were denoted as *x*–Pd/Co_3_O_4_ or Pd/Co_3_O_4_–x; 3% Pd/Co_3_O_4_–Ca (X3), 3% Pd/Co_3_O_4_–Na (X4), 3% Pd/Co_3_O_4_–K (X5), 3% Pd/Co_3_O_4_–Cs (X6), 3% Pd/Co_3_O_4_–Li (X*), 1% Pd/Co_3_O_4_–Li (X7) and 5% Pd/Co_3_O_4_–Li (X8). The % mole of the alkali metal cations was maintained at 2 wt%.

### Catalyst characterization

Various characterization techniques, including ICP-OES, FT-IR, p-XRD, TEM, SEM, TPR, and TPD, were used to characterize the as-prepared nanomaterials. The metal loading (wt%) capacity of the catalyst was determined using the Spectro Arcos ICP-OES instrument. A thermogravimetric analyzer (PerkinElmer STA 6000) was used to perform the thermogravimetric analyses (TGA) under air atmosphere (20–1000 °C) at a flow and heating ramping rates of 20 mL min^−1^ and 10 °C min^−1^, respectively. The surface morphologies of the synthesized nanomaterials were studied using a Vega 3 Tescan LMH Scanning Electron Microscope (SEM) equipped with EDX and operating at a high voltage of 20 kV. The ^1^H NMR (500 MHz) and ^13^C NMR (125 MHz) spectra were acquired using a Bruker-500 MHz spectrometer, with values given relative to tetramethylsilane (0.0) as the internal standard. The N_2_-sorption studies were conducted using a Micromeritics ASAP 2460 device. Prior to the experiments, the samples were degassed at 90 °C under N_2_ atmosphere and vacuum for 14 h and 2 h, respectively, to remove physisorbed moisture from the nanomaterials. The surface area was calculated using the Brunauer–Emmett–Teller (BET) technique. Powder X-ray diffraction (p-XRD) analyses were performed using a Rigaku MiniFlex-600 instrument operating with Cu K radiation (= 1.5406). Both low (0.5–4.0°) and wide (20–70°) 2*θ* angles diffraction patterns were measured at a step rate of 0.1° min^−1^. Fourier transform infrared (FT-IR) spectroscopy of the materials was performed using a Bruker FT-IR Alpha spectrometer (KBr pellets in 4000 to 500 cm^−1^). On a Micromeritics AutoChem II chemisorption analyzer, the hydrogen temperature-programmed reduction (H_2_-TPR) was conducted. About 25 mg of the catalyst was loaded into a quartz tube reactor and pretreated for 1 h at 200 °C under helium/argon flow. After cooling, the materials were probed by passing 10% H_2_ (Ar) at a flow rate of 50 mL min^−1^ from ambient temperature to 600 °C (10 °C min^−1^). The ammonia temperature-programmed desorption (NH_3_-TPD) tests were performed on the Micromeritics AutoChem II equipment to identify the acidic sites on the catalyst. Approximately (0.20–0.30 g) was loaded into a tube reactor containing quartz wool and further probed by passing 10% NH_3_ at a temperature range of 20–700 °C using helium as the probed gas. The flow and heating rates were maintained at 25 mL min^−1^ and 3 °C min^−1^ for both experiments, respectively. The morphology of the catalyst was captured using a Transmission Electron Microscope (TEM) (JEOL Jem-2100F) operated at a 200 kV accelerating voltage. A total of 0.2 mg of the catalyst was measured and ultrasonicated in 1 mL ethanol for 30 min before being deposited on the carbon-coated TEM-Cu grids.

### Catalytic test for carbonylation of the aryl halides

Typically, the reaction tube equipped with a stirring bar was charged with a 0.3 g catalyst (previously reduced at 350 °C for 20 min in H_2_), 4 mmol substrate, 3 mL solvent, 1 eq. Et_3_N B1, 1 mmol (200 L) decane (internal standard), 3 mL HCOOH, and 3 mL nucleophile (for alkoxy- and aminocarbonylation). For 8–24 hours, the mixture was heated to 90–130 °C at a stirring speed of 550 rpm. Upon cooling to RT, 50 mL of water was added to separate the organic phase using a separating funnel. Ethyl acetate was used to extract the aqueous layer (3 × 25 mL). The combined layer was washed with a 50 mL brine solution and then dried over magnesium sulfate. The residual solvent was evaporated using a rotary evaporator, and the product was purified by flash column chromatography on silica gel with hexane/ethyl acetate eluents at various intervals. The products were quantified using a Shimadzu GC-2010 equipped with a flame ionization detector (FID) and N_2_ as the carrier gas; the FID and injection temperatures were adjusted to 370 and 200 °C, respectively. The products were confirmed and compared using GC-MS, ^1^H, and ^13^C NMR instruments. Simple filtration, followed by washing and drying in a vacuum, was used to test the catalyst's reusability.

## Results and discussions

In this study, the newly designed alkali-promoted Pd catalyst was employed as an efficient catalyst for the carboxy- and carbonylation reactions of diverse arrays of aryl halides. Under milder reaction conditions and utilizing formic acid as a CO surrogate source, the carbonylation processes were carried out with excellent conversions, selectivities, and yields to their respective desired products. Most importantly, eliminating the use of acid co-catalyst and sacrificial acetic anhydrides needed for the CO release. In general, this type of carbonylation process is difficult to perform without external additives (organic acids and anhydrides) since these additives are often required to obtain reasonable reactivities.^7,24^ Similarly, homogeneous catalytic systems have been extensively used to carry out these reactions, but with catalyst recovery and reusability problems. As a result, the design of a more versatile heterogeneous catalyst capable of performing these reactions with excellent product yields is highly desired. In this work, we report the first heterogeneous Pd-based catalyst, promoted by alkali metal ions (Cs, Na, K, Li, and Ca) for the carboxy- and carbonylation of aryl halides. Although the PdNPs were the active species for the reaction, however, we found out that the alkali cations in the catalysts had a remarkable promotional effect on the catalytic system by acting as a promoter and an in built cationic Lewis acid co-catalyst. Additionally, the activity was linked with the NH_3_-TPD analysis of each catalyst, showing that the lithium-promoted catalyst with the highest amount of acidic sites was the most active for the reactions (Fig. 4). With the results, we concluded that there was a strong bonding interaction between the alkali cations, palladium nanoparticles, and the mesoporous Co_3_O_4_ support.

### Structural characterization of the catalysts

#### Powder X-ray diffraction (p-XRD)

**Fig. 1:**
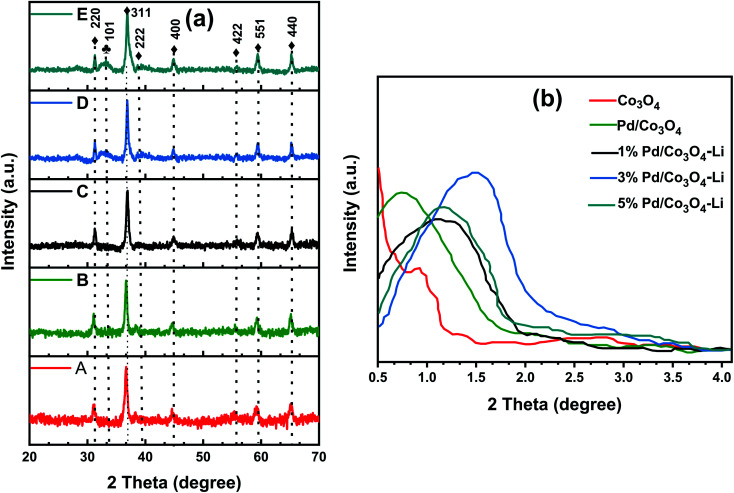
(a) Wide-angle X-ray diffractograms of A (Co_3_O_4_), B (3% Pd/Co_3_O_4_), C (1% Pd/Co_3_O_4_–Li), D (3% Pd/Co_3_O_4_–Li), and E (5% Pd/Co_3_O_4_–Li) and (b) low-angle p-XRD patterns of the nanomaterials. ♦ – Co_3_O_4_-phase (PDF no. 73-1701) and ♣ – PdO-phase (PDF no. 85-0713).

The XRD analysis was used to confirm that the palladium nanoparticles (PdNPs) were effectively immobilized on the support (Co_3_O_4_), and the support's structure was not altered during the synthesis (Fig. 1). Similarly, neither the alkali cations nor the PdNP loadings distorted the spinel Co_3_O_4_ phases. All catalysts exhibited wide-angle diffraction patterns (20–70°) consistent with the spinel Co_3_O_4_ (PDF no. 73-1701) indexed in the JCPDS database; this certifies the absence of other bulk crystalline phases in the nanomaterials. The nanomaterials exhibit low-angle diffraction lines ranging from 0.5 to 4.0°, indicating the existence of a regular ordered mesoporous structure (Fig. 1b). As shown in Table 1, the average size of the nanoparticles grew larger as the Pd loading on the reducible cobalt oxide support was increased. The positions of the low-angle lines and their corresponding shift to smaller 2*θ* values are well correlated with the particle's calculated average size. Notably, a portion of the PdNP was oxidized to PdO as observed from the spectra, which displayed weak diffraction peaks at 33.7°, with the broadest peak assigned to the PdO (101) facet; these peaks were detected for catalysts 3% Pd/Co_3_O_4_–Li (D) and 5% Pd/Co_3_O_4_–Li (E) but were not as prominent for the other catalysts.

**Table 1:** Structural characterization of the nanomaterials

Catalysts	SA_BET_^a^ (m^2^ g^−1^)	Pore volume (cm^3^ g^−1^)	Pore diameter (nm)	Crystallite sizes^b^ (nm)
Co_3_O_4_	63	0.357	12.8	7.4
3% Pd/Co_3_O_4_	58	0.392	19.2	11.6
1% Pd/Co_3_O_4_–Li	51	0.378	16.8	14.2
3% Pd/Co_3_O_4_–Li	43	0.371	15.7	16.8
5% Pd/Co_3_O_4_–Li	29	0.244	10.6	22.4

a Determined by N_2_ BET.

b Calculated using Scherrer's equation.

Additionally, the PdO particles grew in size with increased intensities for the 3 and 5% Pd loadings. However, with a lower Pd loading (1 wt%), no PdO peaks were seen, indicating that the particles were too tiny to be detected by the XRD machine. The peaks corresponding to the Pd metal were not very visible in the spectra. The XRD analysis revealed that the crystallinity of Co_3_O_4_ was preserved, suggesting that the PdNPs and alkali cations were successfully loaded onto its matrix.

#### Scanning/transmission electron microscopy (SEM/TEM)

**Fig. 2:**
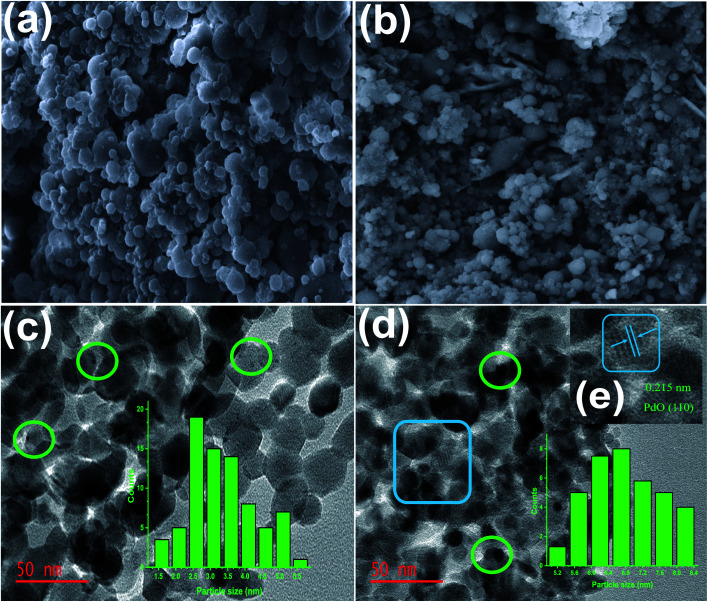
(a) and (b) SEM images of Co_3_O_4_ and 5% Pd/Co_3_O_4_–Li, respectively. TEM images and particle size distribution chart (inset) for (c) 1% Pd/Co_3_O_4_–Li, (d) 5% Pd/Co_3_O_4_–Li and (e) HR-TEM image magnification of 5% Pd/Co_3_O_4_–Li (inset). The nanoparticles are indicated in green circles.

As revealed by the SEM images in Fig. 2a and b, the promoted catalyst exhibits an almost identical ball-like nano-sized shape to Co_3_O_4_, suggesting that the alkali metal and PdNPs did not alter the support's phases. The acquired TEM micrographs show that the size of the PdNPs varies with the amount of Pd loaded on the reducible support (Fig. 2c and d). Irrespective of the Pd loading, the spinel Co_3_O_4_ particles display a similar size around 25–40 nm. A low population of the PdNPs on Co_3_O_4_ was detected for the 1% loading and grew larger for the 5% loading; this directly correlates with and is consistent with the XRD data. The 0.215 nm lattice fringes found for 5% Pd/Co_3_O_4_–Li, can be indexed to the {110} planes of PdO (Fig. 2e).^50,51^

#### Nitrogen sorption studies

**Fig. 3:**
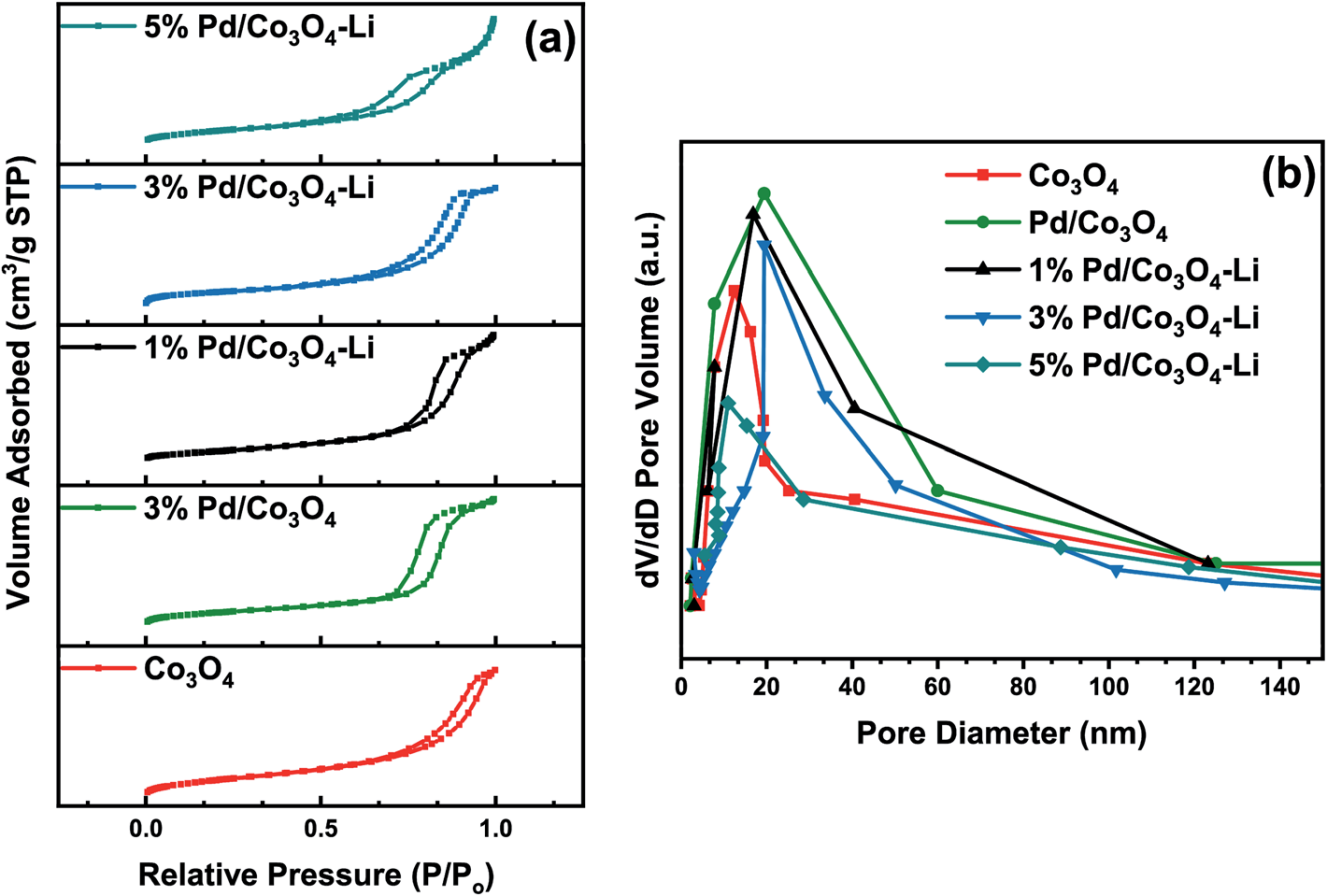
(a) Nitrogen adsorption–desorption curves and (b) BJH pore size distributions for the catalysts.

To study how the Pd size varied with the loadings, the surface area, pore size, and pore volume were determined using BET analysis. As shown in Fig. 3a, the sorption curves of the nanomaterials exhibited a type IV isotherm with minor distortions and a relative pressure range of 0.76–0.92, confirming the material's mesoporous structure. It is noteworthy that increasing the Pd loading decreased the surface area (SA), pore-volume, and pore diameter, with the least observed for the 5% Pd loading, suggesting that Pd loadings had a significant effect on the pore structures and catalyst's SA. The decrease in SA, pore volume/diameter, was most probably caused by PdNPs occupying a portion of the Co_3_O_4_ mesopores. In many catalytic reactions, catalytic performance is often ascribed to the physiochemical properties of the catalyst, such as surface areas, pore sizes/volumes.^52–57^ It is critical to highlight that these features were not overt or considered a determining factor in the moderate to high catalytic activity reported for the nanomaterials in this study. The textural properties of nanoparticles are summarized in Table 1.

#### Temperature-programmed measurements

**Fig. 4:**
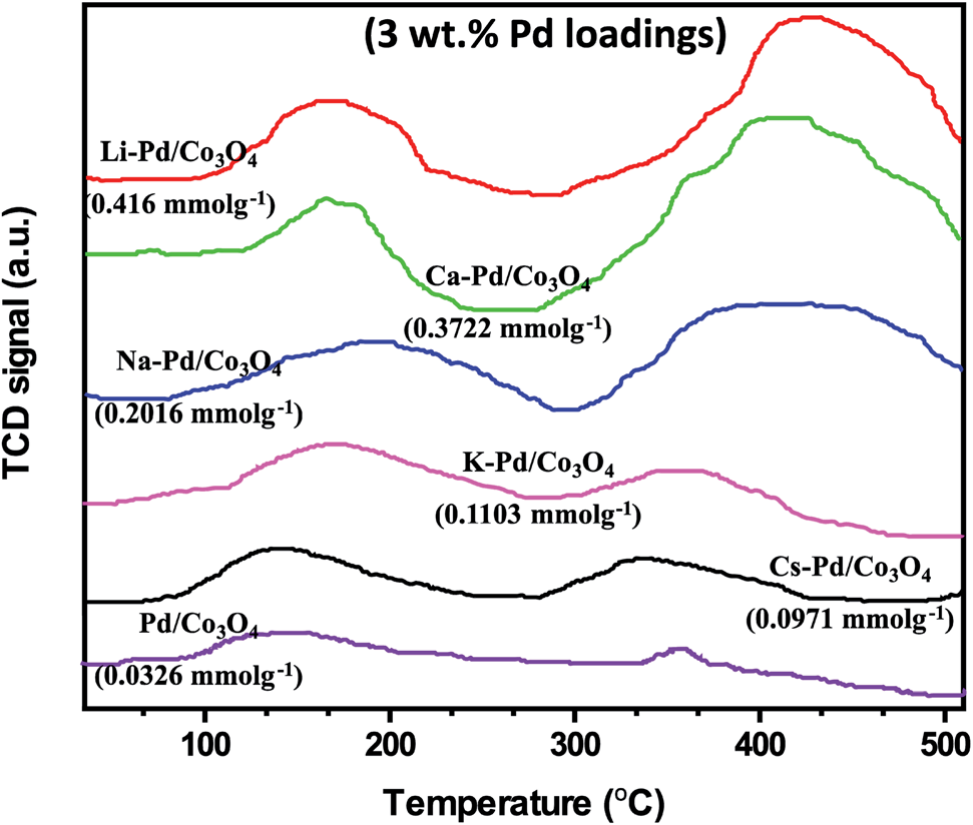
NH_3_-TPD profiles of the catalysts. All Pd loadings were set at 3 wt%.

The ammonia temperature-programmed desorption (NH_3_-TPD) measurement was used to determine the acidic sites of promoted and unpromoted catalysts, and their representative spectra/total acidity amount (mmol of NH_3_ per gram of catalyst) are presented in Fig. 4. Compared to the other catalysts, the Li-promoted catalyst showed a more prominent and robust acidity site (0.4160 mmol g^−1^) with a shoulder peak at 438 °C. This phenomenon may be ascribed to the highly electropositive alkali cations in the catalyst, which increased the acid strength and enhanced the catalytic activity for the reactions. The unpromoted Pd/Co_3_O_4_ catalyst exhibited the least acidic sites of all the catalysts analyzed, with a peak around 352 °C. It is important to note that the increase in acidity was found to correlate with the alkali metal ions' cationic Lewis-acid strength (Li > Ca > Na > K > Cs).

**Fig. 5:**
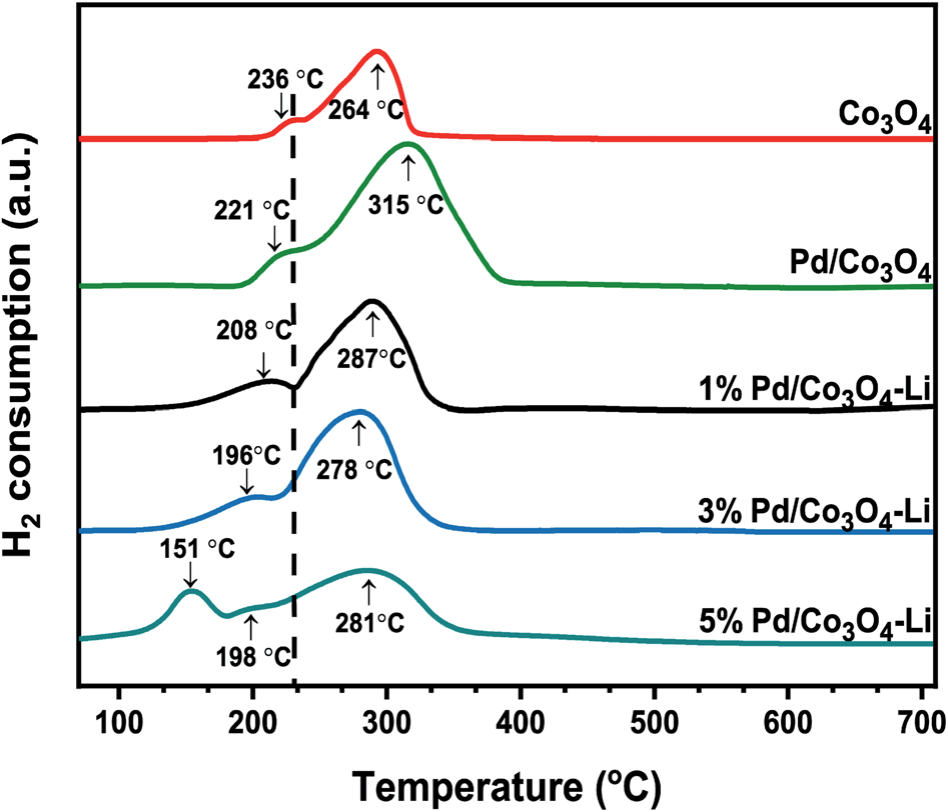
H_2_-TPR profiles of the catalysts.

Following that, we conducted the H_2_-TPR measurements to determine the nanomaterials' reducibility (Fig. 5). Two reduction peaks at 236 °C and 264 °C, assigned to Co^3+^ → Co^2+^ and Co^2+^ → Co, respectively, were observed for the mesoporous Co_3_O_4_. The addition of palladium and an alkali cation to Co_3_O_4_ facilitated the catalyst's reduction behavior, consistent with previous reports.^58–60^ The Pd loadings substantially influenced the shift to lower reduction temperatures; these peaks at lower temperatures were attributed to PdO reductions, and the peak positions are highly dependent on the PdO dispersion on the support.^61^ Although the low-temperature peaks may not be entirely due to PdO reduction, because catalysts have been shown to display a reduction of Co^3+^ → Co^2+^ at temperatures as low as 180–200 °C, apparently due to the H_2_ spillover effect.^61–63^ However, the lower reduction peak in the range of 151–196 °C for the 3% X* and 5% X8 catalysts may be attributed to PdO reduction, as this was also evident from the XRD spectra. It is worth noting that the trend toward increasing catalytic activity of the catalyst may also be linked to the nanomaterial's low reduction temperatures.

#### Thermogravimetric (TGA) and Fourier-transform infrared (FT-IR) spectrometry

The thermographic analysis curves for the nanomaterials are depicted in Fig. S2 and S3.† This analysis was used to validate the thermal stability of the as-prepared catalysts over a temperature range of 0–1000 °C. The spectra revealed a minor and significant weight loss between 160 and 1000 °C, ascribed to absorbed moisture on the catalyst surface (160–200 °C) and thermal degradation of the organic/inorganic matter (206–800 °C). All catalysts exhibited peaks associated with metal bands in their FT-IR spectra. It is worth mentioning that the Pd and alkali-cations did not alter the structure of the mesoporous Co_3_O_4_ (Fig. S4†).

### Catalytic reactions

#### Catalyst screening

**Fig. 6:**
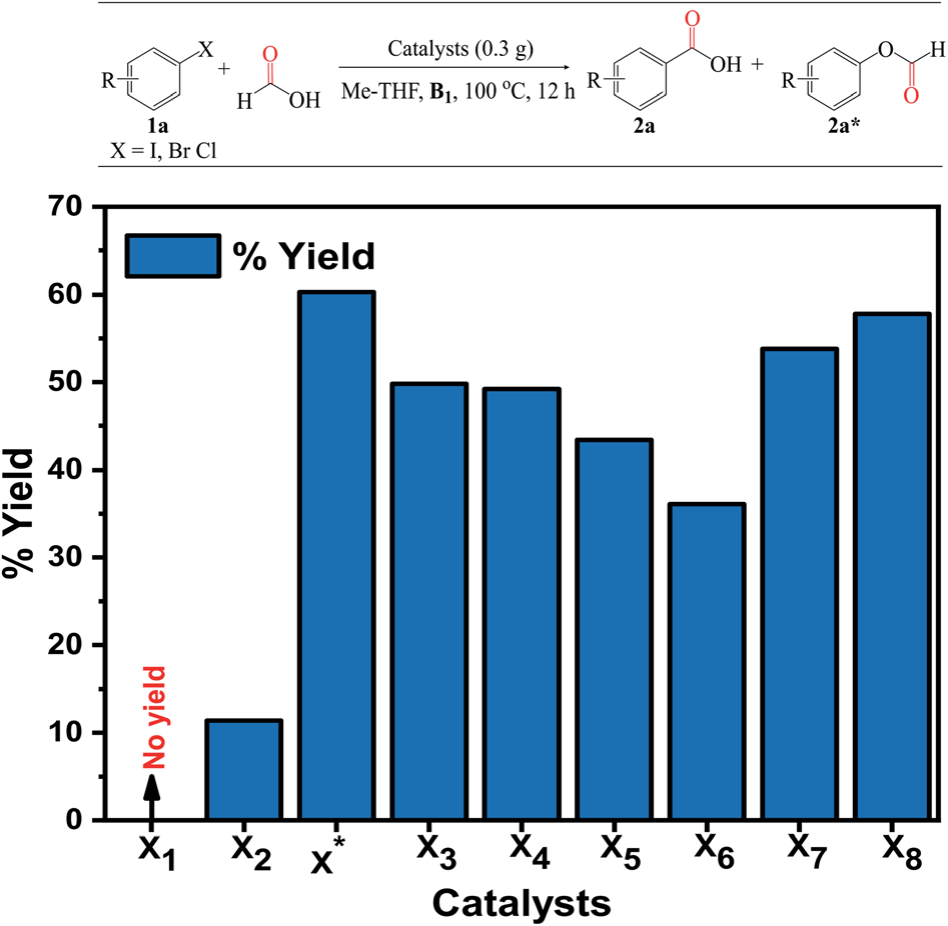
Catalyst screening. Reaction conditions: catalyst (0.3 g), 1a (4 mmol), HCOOH : Me-THF 3 : 3 (mL), B1 (1 equiv.), 100 °C, 12 h. Isolated yield and selectivity (%) determined by GC-FID analysis.

The carboxylation reaction was used to establish the general concept for the newly developed alkali-promoted Pd/Co_3_O_4_. We focused our attention on bromobenzene 1a as a model substrate. The reactions were carried out in the absence of toxic/flammable organic acids, and anhydrides (activators). To begin, we screened the catalysts, Co_3_O_4_ (X1), 3% Pd/Co_3_O_4_ (X2), 3% Pd/Co_3_O_4_–Ca (X3), 3% Pd/Co_3_O_4_–Na (X4), 3% Pd/Co_3_O_4_–K (X5), 3% Pd/Co_3_O_4_–Cs (X6), 3% Pd/Co_3_O_4_–Li (X*), 1% Pd/Co_3_O_4_–Li (X7) and 5% Pd/Co_3_O_4_–Li (X8). As shown in Fig. 6, the reaction did not occur in the absence of the Pd and alkali metal ions for the X1 catalyst. When Pd was added to X1 to give X2, an improvement in the 2a product yield of 12.4% was recorded, which rose significantly to 36.1–60.3% following doping with the alkali metal ions (x), suggesting that the alkali metal ions are essential to achieve reasonable 1a conversion. Gratifyingly, it was discovered that the lithium-promoted catalyst X* was the most active to the others, with a moderate selectivity and yield of 60.3% obtained. An increase in the Pd loading up to 5% (X8) had no discernible effect on the 2a yield (57.8%), and likewise when the Pd loading was reduced to 1% (X7). Meanwhile, the 2a* byproduct (phenyl formate) formed was as a result of a possible nucleophilic attack of HCOOH, which resulted in a lower yield for some of the reactions. According to the activity trend, Pd is the active species in these reactions, while the alkali-cations act as promoters/co-catalysts and are critical for stabilizing the active Pd species on the support. The increase in the catalytic activity of the Li-promoted catalyst was proportional to the cationic Lewis-acid strength, in the order Li > Ca > Na > K > Cs and may be attributed to the lithium's high charge density and low ionic radii, which promoted and triggered the Pd species' charge site for the reactions to proceed smoothly.

#### Optimization of reaction conditions

With the best catalyst X* in hand, we reported the optimization of reaction conditions. First, we investigated several reaction parameters (catalyst loading, time, temperature, solvents, bases, and nucleophiles) under optimized reaction conditions. The results are summarized in Table 2. When the catalyst amount was reduced to 0.1 g, the yield 2a decreased significantly to 47.4% (Table 2, entry 2). At 8 h reaction time, the conversion and yield decreased to 52.8% and 36.8%, respectively (Table 2, entry 4), and increased slightly to 66.2% after 16 h (Table 2, entry 5). After twelve hours, when the reaction temperature was raised to 110 °C, the conversion and yield rose substantially to 85.5 and 76.2%, respectively (Table 2, entry 6). At 120 °C, an increase of 93.6% in 1a conversion was observed (Table 2, entry 7). The reaction was not favorable for temperatures <80 °C, with the conversion and yield recorded at <20% (Table 2, entry 8). Next, we focused on utilizing some selected conventional organic solvents with the reaction conditions: 110 °C, 12 h. As expected, the solvents (Table 2, entries 9–11) provided significant yields and selectivities, with the highest yield still obtained for Me-THF (76.2%) (Table 2, entry 6); this is presumably due to its moderate polarity, which enables it to dissolve a broad range of organic compounds. Between K_2_CO_3_ and the triethylamine, significant substrate conversions were obtained for both bases, indicating that the triethylamine, a versatile base used in carbonylation reactions, can be conveniently replaced with a non-volatile base (K_2_CO_3_) (Table 2, entry 12).

**Table 2:** Effect of reaction parameters^a^

Entry	Cat. X* (g)	Time (h)	Temp. (°C)	Solvents	Nucleophiles	Conv.^b^ (%)	Sel.^b^ (%)	Yield^b^ (%)
1	0.3	12	100	Me-THF	—	75.1	80.4	60.3
2	0.1	12	100	Me-THF	—	66.3	71.3	47.4
3	0.5	12	100	Me-THF	—	79.9	76.8	61.7
4	0.5	8	100	Me-THF	—	52.8	70.4	36.8
5	0.5	16	100	Me-THF	—	81.8	79.6	66.2
6	0.5	12	110	Me-THF	—	85.5	90.2	76.2
7	0.5	12	120	Me-THF	—	93.6	74.7	71.1
8	0.5	12	70	Me-THF	—	<20	—	<20
9	0.5	12	110	Acetonitrile	—	70.7	65.3	45.9
10	0.5	12	110	1,4-Dioxane	—	90.5	67.1	59.8
11	0.5	12	110	Toluene	—	85.5	70.3	59.5
12^c^	0.5	12	110	Me-THF	—	80.6	38.2	30.6
13	0.5	12	110	—	Methanol	85.3	57.2	48.7
14	0.5	12	110	—	Ethanol	75.2	51.9	39.1
15	0.5	12	110	—	Propanol	68.4	52.4	35.9
16	0.5	12	110	—	Butanol	75.3	44.2	33.2
17	0.5	12	110	—	*tert*-Butanol	60.9	38.5	23.5
18	0.5	12	110	—	Cl-butanol	69.3	50.5	35.1

a Reaction conditions: 3% X* (0.1–0.5 g), 1a (4 mmol), HCOOH : nucleophiles : Me-THF 3 : 3 : 3 (mL), B1 (1 equiv.).

b Conversion, selectivity and isolated yields determined by GC-FID.

c (X*, 0.5 g) with K_2_CO_3_ (1 equiv.). Entries 1–12 (carboxylation). Entries 13–18 (alkoxycarbonylation).

Additionally, to demonstrate the versatility of this novel catalyst under the optimized reaction conditions, we validated the possibility of using different nucleophiles to investigate these alkyl group's effects on the alcohol for the alkoxycarbonylation reaction. Notably, methanol was the most efficient nucleophile compared to the others, though, no significant difference in product yields and selectivities (Table 2, entries 13–18). The observation thus implies that an increase in the alkyl chain length on the alcohols may substantially reduce yields owing to the formation of additional side products. The yield of 2a dropped to 23.5% when *tert*-butanol was used, presumably due to steric hindrance from the bulk methyl substituents attached to the alcohol molecule (Table 2, entry 17). Interestingly, the yield of the chloro-substituted butanol significantly increased compared to its unsubstituted counterpart, demonstrating the effect of the electron-withdrawing group on the nucleophile (Table 2, entry 18).

#### General substrate scope for the reaction

**Fig. 7:**
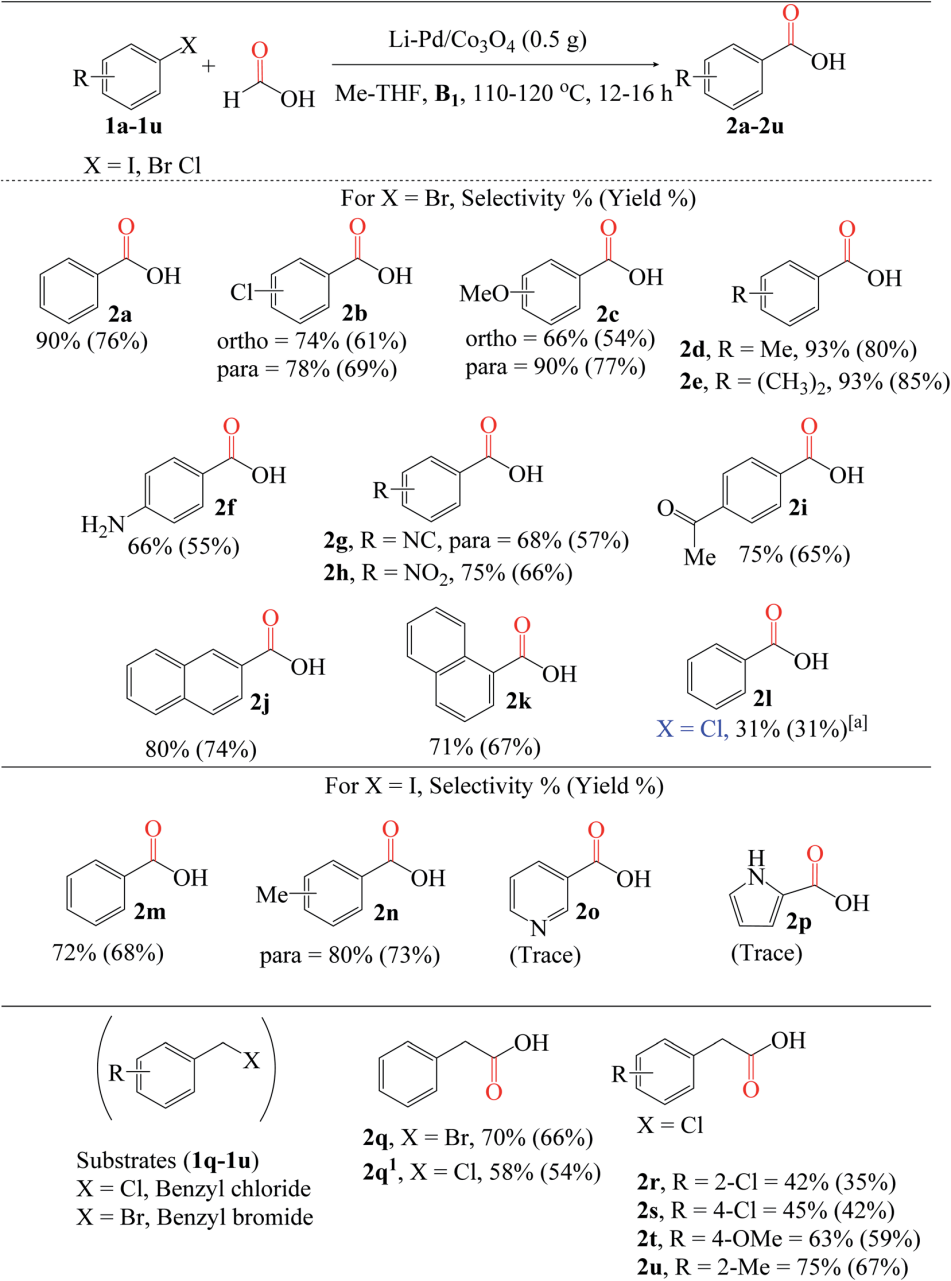
Reaction conditions: 3% X* (0.5 g), substrates (4 mmol), HCOOH : Me-THF 3 : 3 (mL), B1 (1 equiv.). Conversion and isolated yields determined by GC-FID analysis. ^*a*^Reaction conditions: 5% X* (1.0 g), 1l (4 mmol), HCOOH : Me-THF 6 : 6 (mL), B1 (1 equiv.), 130 °C, 24 h.

To further explore the flexibility of this new catalyst, we investigated a variety of aryl halides (Fig. 7). Interestingly, carboxylation of various substituted aryl halides (1a–1n) produced carboxylic acid products with moderate to excellent yields (2a–2n). Notably, the halo group attached to the aryl bromides, such as 1b (Cl), survived the reaction regardless of substitution mode. In terms of general applicability, aryl bromide substrates containing electron-donating groups, such as methoxy 1c, *para*-methyl 1d, *para*-substituted dimethyl 1e, and amino 1f, and electron-withdrawing groups (cyano 1g, nitro 1h, and carbonyl 1i), were found to be compatible with the reaction, yielding moderate to excellent 2c–2i products. Furthermore, we extended the protocol to the 2-iodonapthalene substrate 1j and its sterically hindered counterpart 1k. Notably, the former provided a much better 2j yield than the latter (2k). Following that, we screened the 1l chlorobenzene (aryl chloride) substrate; unfortunately, a yield of <10% was obtained. Despite significant progress made over the last decades,^64^ carbonylation of non-activated chlorobenzene and its derivatives to their desired products remains a challenge compared to iodo- and bromobenzene. This is due to the higher CO pressure and temperature required to achieve reasonable conversion, owing to the molecule's robust C(sp^3^)–Cl bonds. However, a reasonable 2l yield of up to 31% was obtained by increasing the Pd loading/catalyst amount, temperature, and time. Carboxylation of the aryl iodide substrates (1m and 1n) was well-tolerated. The reaction, however, was incompatible with aryl iodides containing pyridine moieties (1o and 1p). Only trace amounts of 2o and 2p yields were obtained.

Besides aryl iodides and bromides, benzyl halides (BnCl and BnBr) can also be converted to carboxylic acids. Comparing the reactivities of 1q (BnBr) and 1q^1^ (BnCl), the former yielded much more 2q than the latter (2q^1^), at 66% and 54%, respectively; this is directly related to and consistent with the relative stabilities of the C(sp^3^)–X bonds. Regardless of the substitution position, moderate yields of 2r (2-Cl) and 2s (4-Cl) were obtained using chloro-substituted benzyl chloride (1r and 1s). Similarly, electron-donating groups (OMe and Me) attached to the benzyl chloride substrates 1t and 1u were effectively carboxylated to 2t (59%) and 2u (67%), respectively.

**Fig. 8:**
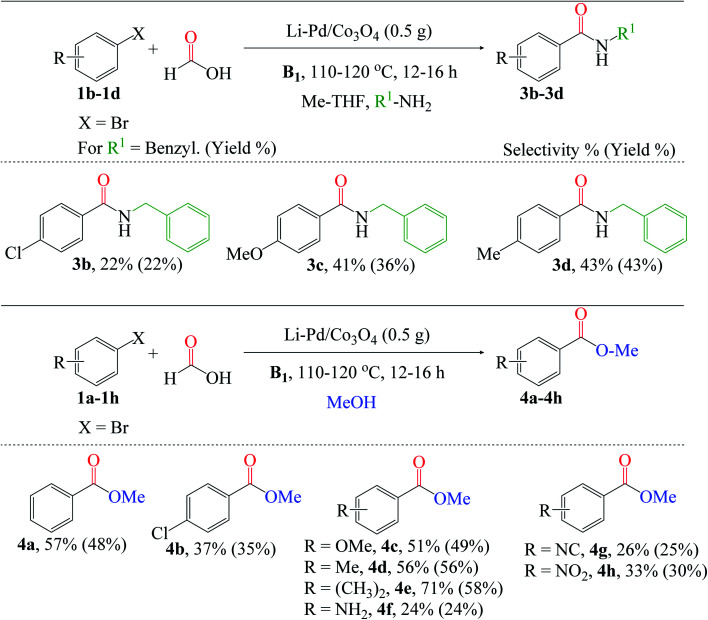
Reaction conditions: 3% X* (0.5 g), substrates (4 mmol), HCOOH : MeOH : R^1^-NH_2_ : Me-THF 3 : 3 : 3 : 3 (mL), B1 (1 equiv.). Conversion and isolated yields were determined by GC-FID analysis.

Additionally, we broadened the substrate scope for amino- and alkoxycarbonylation of aryl halides (Fig. 8). Since these reactions are relatively the same, we also utilized the optimized reaction conditions stated above in Fig. 7. The aminocarbonylation of *p*-chlorobromobenzene 1b was catalyzed by X* using benzylamine as the nucleophile. Electron-donating group substrates, such as 1c (MeO) and 1d (Me), were successfully utilized to obtain the desired amides (3c and 3d). Also, the chlorosubstituted 1b substrate was tolerated. Next, we explored the versatility of the catalyst for the alkoxycarbonylation reaction. The chlorosubstituted bromobenzene 1b was successfully alkoxycarbonylated to their corresponding ester 4b in moderate yield. Interestingly, excellent yields of 4c–4h were obtained using substrates substituted with electron-donating groups such as 1c (OMe), 1d (Me), 1e (CH_3_)_2_, and 1f (NH_2_), as well as electron-withdrawing groups such as 1g (NC) and 1h (NO_2_). Lastly, it is worth noting that the alkoxycarbonylation of 1d was completed in a shorter reaction time than the aminocarbonylation reaction, demonstrating the substrate's high reactivity for the alkoxycarbonylation reaction.

### Kinetic and GC-TCD studies

**Fig. 9:**
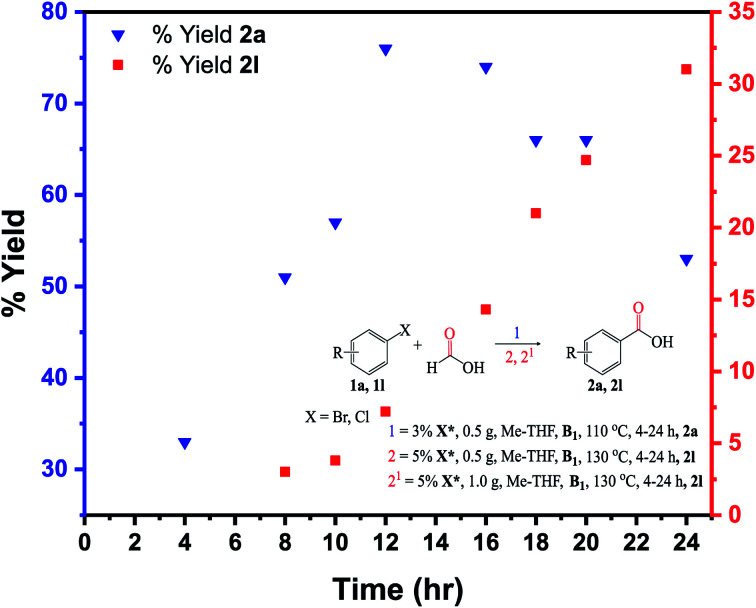
Reaction kinetics for the 1a and 1l substrates: isolated yields determined by GC-FID analysis. Under the same reaction conditions (110 °C, 8 h), the 1l substrate recorded a yield of 2l at <5%. At 5% X* (1.0 g), 130 °C, 24 h, the 2l yield reached >30%.

The kinetic analysis was carried out on the substrates 1a (bromobenzene) and 1l (chlorobenzene) (Fig. 9). Carboxylation of 1a was much faster than 1l at a 8 h reaction time, with conversion and yield to the target product 2a reaching 73% and 51%, respectively. Conversion and yield of the 1l substrate were both <5% under identical reaction conditions. When the time was extended to 20 h, the conversion of 1a reached almost 100%, while the complete conversion of 1l took around 24–30 h. The kinetic study implies that a shorter time (low pressure) is required for carboxylation of the 1a, while a longer time (higher pressure/temperature) is needed for carboxylation of the 1l due to the stronger C(sp^3^)–Cl bonds in the 1l molecule (Fig. 9).

To gain a better insight into the formic acid decomposition of this new system, a GC-TCD analysis was performed (Fig. S5†). Several literature works have reported the use of acid anhydrides and other activators to facilitate the CO release from formic acid.^19,65,66^ Despite the moderate to good yields obtained with these additives, product contamination has been associated with this protocol due to the anhydride's slow decomposition and severe reaction conditions, resulting in a longer time required to stabilize the catalytic system. Additionally, it is well established that the same acid anhydrides are incompatible with aminocarbonylation reactions due to the formylating agents (acetic formic anhydride) formed in solution during the CO release process.^67^ This necessitates the development of safer procedures for this type of reaction. Typically, an autoclave equipped with a stirring bar was charged with X* (0.5 g), HCOOH (3 mL), Me-THF (3 mL), and B1 (1 eq.) and vigorously stirred at 550 rpm for 16 h (110–130 °C). The reaction was halted and allowed to cool to ambient temperature before being analyzed on the GC-TCD machine.

Interestingly, the formic acid decomposed into a mixture of CO and CO_2_, with the highest amount/area obtained for the CO, suggesting that this system can readily be used *in lieu* of acid anhydrides for the *in situ* generation of CO from formic acid. Although, a minute amount of H_2_ gas was also detected in the spectra after 3.436 retention time. It is worth noting that the new catalyst serves a dual purpose: it facilitated the decomposition of the HCOOH and triggers the substrates' C(sp^3^)–X bonds, allowing for clean carbonylative transformations.

Additionally, an open-system experiment was conducted to prove the *in situ* generation of CO (Fig. S6†). The 2a yield decreased substantially under this “open-system condition” compared to the conventional procedure described before, probably due to the reduced gas pressure released throughout the process. As a result, the experiment verifies that the generated gas is CO (see Fig. S6 in the ESI†).

### Reusability and heterogeneity test

**Fig. 10:**
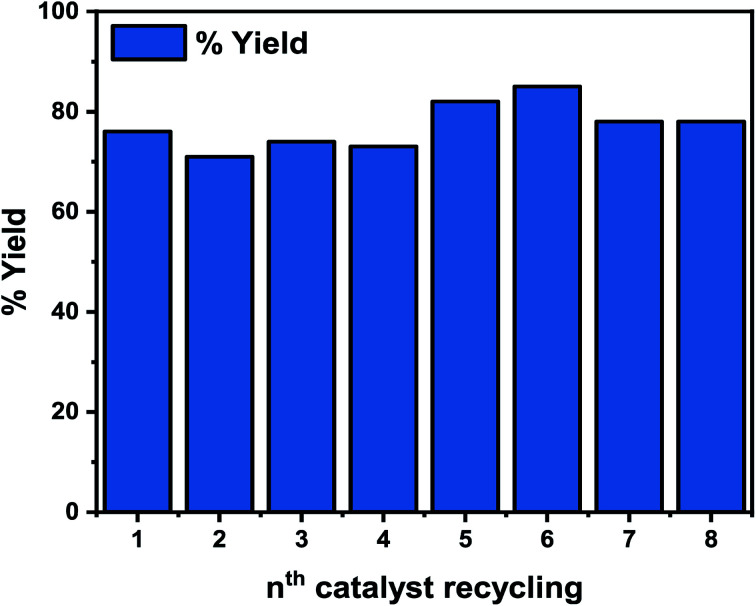
Catalyst reusability. Reaction conditions: 110 °C, 12 h.

The reusability of the Pd-based heterogeneous catalyst was tested with the model reaction (carboxylation) using our benchmark substrate 1a, under the optimized reaction conditions of 110 °C, 12 h (Fig. 10). Centrifugation at 450 rpm for 30 min was used to separate the catalyst, followed by simple filtration and reactivation in an oven at 350 °C for 30 min before reuse. As shown in Fig. 10, the 2a yield decreased in the second, third, and fourth cycles but significantly rose in the fifth and sixth cycles, reaching 82 and 85%, respectively, indicating its stability. Additionally, our initial test reveals that the unpromoted catalyst exhibited significant leaching of the active Pd in the reaction mixture. The increased stability of the Li-promoted catalyst over its unpromoted counterpart may be attributed to the alkali metal in the catalyst, which minimized leaching of the active Pd species, improved the catalyst's binding properties, and enhanced the catalytic activity for the reactions (Fig. 10). It is worth noting that the reusability of thi novel catalyst significantly improves the viability of our catalytic system in comparison to the homogeneous systems.^68–72^

Next, the heterogeneity test was performed by first filtering the composite catalyst from the reaction mixture, and after that, the filtrate was reintroduced into the reaction tube under the reaction conditions of 110 °C and 8 h. Interestingly, no change in conversion was observed after 8 h of reaction time (Fig. S7†), suggesting that the active Pd species that catalyzed the reaction was not leached into the mixture. In addition, the ICP-OES analysis of the filtrate was performed after the 8^th^ run, with no possible bleeding of the Pd species.

## Conclusion

The carboxy- and carbonylation reactions were achieved with the newly designed heterogeneous Pd catalyst and synthetic protocol, which *in situ* generates CO from formic acid. Although the system was more favorable for carboxylation reaction, acceptable product yields were also obtained for alkoxy- and aminocarbonylation processes. Without the need for acid co-catalysts and acetic anhydrides, the reactions proceeded smoothly with this new catalyst under mild reaction conditions and at a shorter reaction time. Besides the catalytic system's superior selectivity and unprecedented reactivity, another more exciting feature was observed in the catalyst's practical reusability. We believe that the reported results adequately demonstrate the synthetic utility of this green carbonylation protocol and therefore establish it as a critical tool for development in academia, the chemical industry, and drug delivery applications.

## Conflicts of interest

There are no conflicts of interest to declare.

## Supplementary Material

RA-011-D1RA05177F-s001
