# Supplementary material for: Alkali-modified heterogeneous Pd-catalyzed synthesis of acids, amides and esters from aryl halides using formic acid as the CO precursor
Source: RSC Adv. 2021 Aug 5;11(43):26937–48. doi: 10.1039/d1ra05177f (PMC9037739; doi:10.1039/d1ra05177f)
Supplement: RA-011-D1RA05177F-s001 [file RA-011-D1RA05177F-s001.pdf]

Electronic Supplementary Material (ESI) for RSC Advances.
This journal is © The Royal Society of Chemistry 2021

Supplementary information

Alkali-modified heterogeneous Pd-catalyzed synthesis of acids, amides and esters from aryl halides using formic acid as the CO precursor

Charles O. Oseghale, Oluwatayo Racheal Onisuru, Dele Peter Fapojuwo, Batsile M. Mogudi, Pule Petrus Molokoane, Nomathamsanqa Prudence Maqunga and Reinout Meijboom*

Research Center for Synthesis and Catalysis, Department of Chemical Sciences, University of Johannesburg, PO Box 524, Auckland Park 2006, Johannesburg, South Africa Tel.: +27 (0)72 894 0293, Fax: +27 (0)11 559 2819
Corresponding Author* E-mail: rmeijboom@uj.ac.za (R.M.)

General Considerations

Unless otherwise stated, all chemicals were purchased from Sigma-Aldrich and Associated Chemical Enterprises and used as received. The ^{1}H NMR (500 MHz) and ^{13}C NMR (125 MHz) spectra were recorded on a Bruker-500 MHz spectrometer, with reported values relative to tetramethylsilane (δ 0.0) as the internal standard. Multiplets were assigned as singlet (s), doublet (d), triplet (t), quartet (q) and double doublet (dd). Unless otherwise stated, isolated yields were determined by ^{1}H-NMR spectroscopy and GC equipped with FID. All other measurements were performed at room temperature unless otherwise stated.

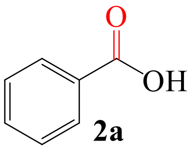

2a

Isolated yield: 76%

^{1}H NMR ($CDCl_3$, 500 MHz): δ 8.71 (dddd, J = 8.2, 1.7, 1.3, 0.3 Hz, 2H), 7.57 (tt, J = 7.2, 1.3 Hz, 1H), 6.42 (dddd, J = 8.2, 7.3, 1.2, 0.3 Hz, 2H); ^{13}C NMR ($CDCl_3$, 125 MHz): δ 178.2, 149.5, 146.6, 146.9, 145.8.

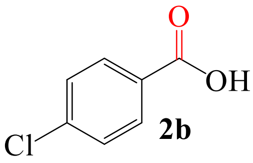

2b

Isolated yield: 69%

^{1}H NMR ($CDCl_3$, 500 MHz): δ 8.21 (ddd, J = 8.4, 1.3, 0.3 Hz, 2H), 6.57 (ddd, J = 8.4, 1.3, 0.3 Hz, 2H); ^{13}C NMR ($CDCl_3$, 125 MHz): δ 178.3, 165.3, 161.2, 148.5, 143.6.

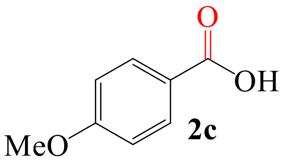

2c

Isolated yield: 77%

^{1}H NMR ($CDCl_3$, 500 MHz): δ 8.41 (dd, J = 8.3, 1.3 Hz, 2H), 8.22 (dd, J = 8.3, 1.1 Hz, 2H), 3.36 (s, 3H); ^{13}C NMR ($CDCl_3$, 125 MHz): δ 178.8, 169.3, 149.2, 138.7, 136.3, 59.1, 57.2, 55.1.

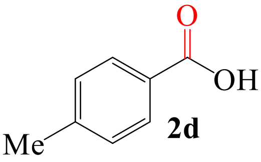

2d

Isolated yield: 80%

^{1}H NMR ($CDCl_3$, 500 MHz): δ 7.81 (dd, J = 8.2, 1.5 Hz, 2H), 7.51 (dd, J = 8.2, 1.3 Hz, 2H), 2.62 (s, 3H); ^{13}C NMR ($CDCl_3$, 125 MHz): δ 173.5, 142.4, 141.7, 138.2, 22.6, 16.9.

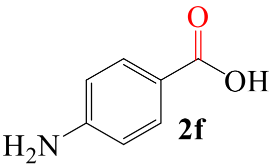

2f

Isolated yield: 55%

^{1}H NMR ($CDCl_3$, 500 MHz): δ 8.28 (dd, J = 8.2, 1.5 Hz, 2H), 7.21 (dd, J = 8.2, 1.3 Hz, 2H), 2.46 (s, 3H); ^{13}C NMR ($CDCl_3$, 125 MHz): δ 175.2, 157.9, 153.4, 143.5, 35.9.

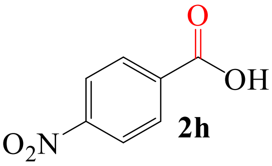

2h

Isolated yield: 66%

^{1}H NMR ($CDCl_3$, 500 MHz): δ 7.35 (dd, J = 8.2, 1.5 Hz, 2H), 7.24 (dd, J = 8.2, 1.3 Hz, 2H), 2.52 (s, 3H); ^{13}C NMR ($CDCl_3$, 125 MHz): δ 177.9, 159.4, 157.2, 143.6, 122.3, 118.5, 39.3.

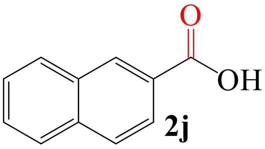

2j

Isolated yield: 74%

^{1}H NMR ($CDCl_3$, 500 MHz): δ 7.71 (ddq, J = 2.0, 1.7, 0.4 Hz, 1H), 7.40-7.46 (dddt, J = 7.5, 1.7, 1.6, 0.4 Hz, 2H), 7.23 (ddd, J = 8.3, 2.0, 0.4 Hz, 2H), 7.06-7.18 (dddd, J = 7.5, 6.9, 2.5, 0.4 Hz, 2H); ^{13}C NMR ($CDCl_3$, 125 MHz): δ 176.3, 144.5, 143.2, 137.6, 136.7, 114.7.

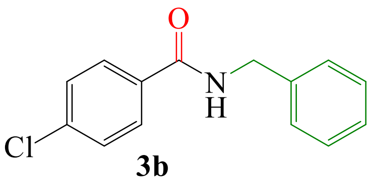

3b

Isolated yield: 22%

^{1}H NMR ($CDCl_3$, 500 MHz): δ 8.45 (ddd, J = 8.4, 1.7, 0.4 Hz, 2H), 8.27 (ddd, J = 8.4, 1.2, 0.4 Hz, 2H), 7.67-7.84 (dddd, J = 7.3, 1.2, 1.0, 0.4 Hz, 5H, tdd, J = 7.4, 1.7, 0.4 Hz), 4.18 (s, 2H); ^{13}C NMR ($CDCl_3$, 125 MHz): δ 176.6, 148.1, 141.3, 134.3, 132.7, 40.2.

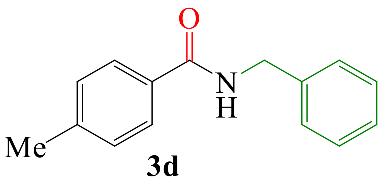

3d

Isolated yield: 43%

^{1}H NMR ($CDCl_3$, 500 MHz): δ 8.17 (ddd, J = 8.2, 1.6, 0.4 Hz, 2H), 7.53-7.75 (dddd, J = 7.3, 1.2, 1.0, 0.4 Hz, 5H, tdd, J = 7.4, 1.7, 0.4 Hz, tt, J = 7.3, 1.2 Hz), 2.47 (s, 3H); ^{13}C NMR ($CDCl_3$, 125 MHz): δ 174.1, 146.7, 141.2, 138.1, 132.1, 129. 1, 58.2, 31.3.

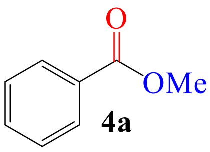

4a

Isolated yield: 48%

^{1}H NMR ($CDCl_3$, 500 MHz): δ 8.74 (dddd, J = 8.2, 1.7, 1.3, 0.2 Hz, 2H), 7.72 (tt, J = 7.3, 1.2 Hz, 1H), 7.43 (dddd, J = 8.2, 7.0, 1.2, 0.3 Hz, 2H), 4.18 (s, 3H); ^{13}C NMR ($CDCl_3$, 125 MHz): δ 174.5, 130.3, 52.5.

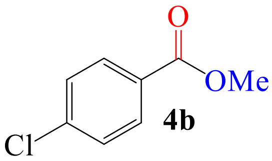

4b

Isolated yield: 35%

^{1}H NMR ($CDCl_3$, 500 MHz): δ 7.64 (dd, J = 8.2, 1.3 Hz, 2H), 7.37 (dd, J = 8.2, 1.3 Hz, 2H), 3.76 (s, 3H); ^{13}C NMR ($CDCl_3$, 125 MHz): δ 172.3, 162.3, 132.9, 130.8, 56.4.

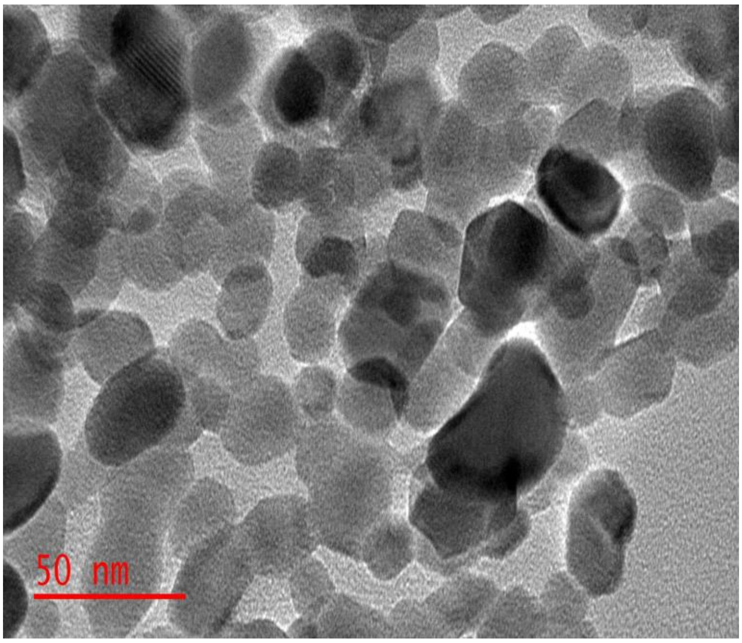

Fig. S1 TEM image of Co_3O_4.

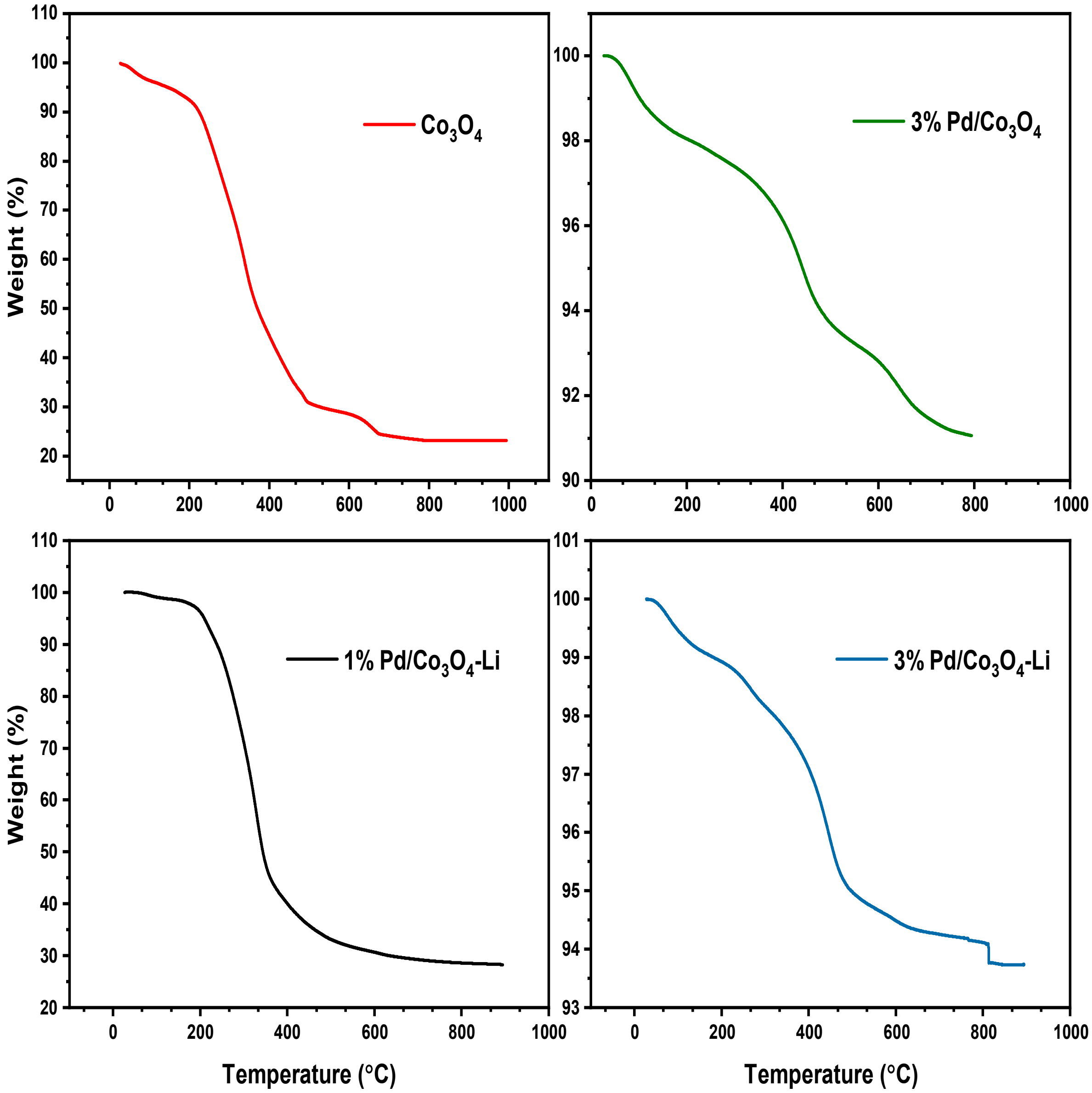

Fig. S2 TGA spectra of the catalyst.

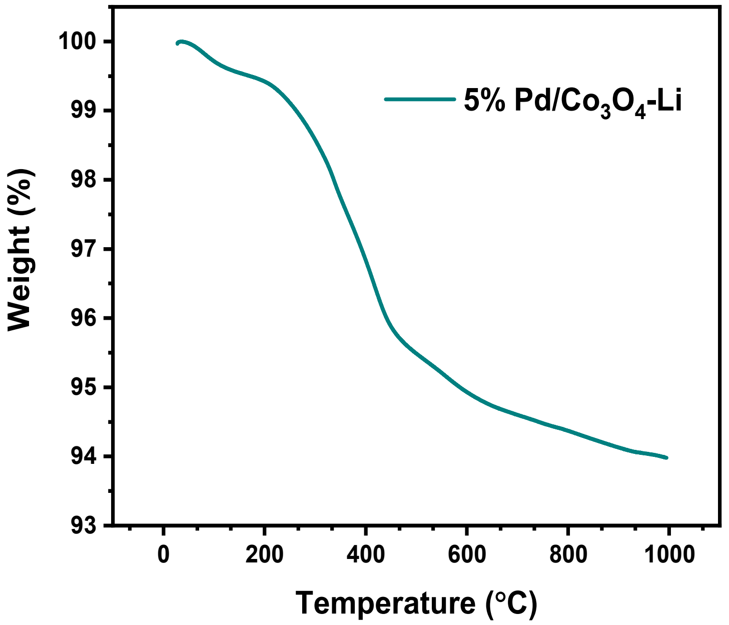

Fig. S3 TGA spectra of the catalyst.

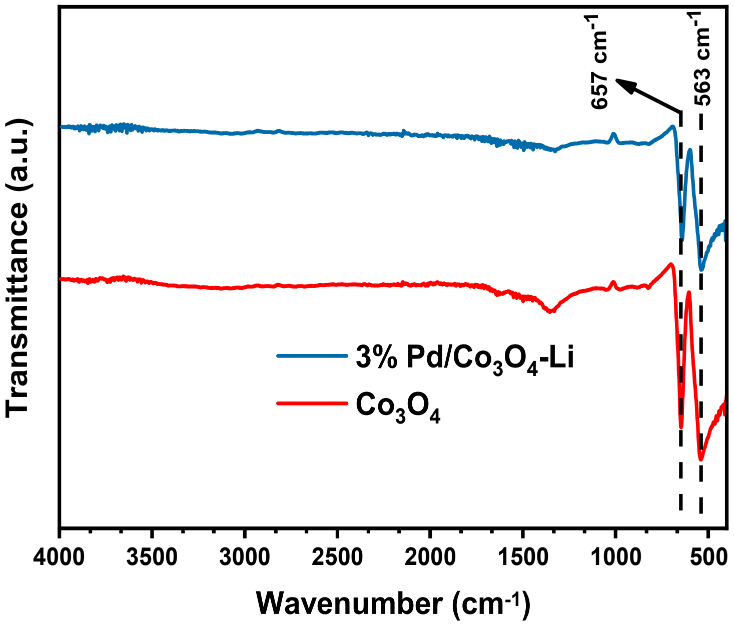

Fig. S4 FT-IR spectra of the catalyst.

Lab name: SRI Instruments
Client: Valued Customer
Client ID: N2024
Analysis date: 07/22/2021 11:19:04
Method: Syringe Injection
Description: TCD
Column: RESTEK 15METER MXT-1
Carrier: HELIUM AT 5 PSI
Integration: Peak sens=95.0 Base sens=60.0 Min area= 100.00 Standard= 1.00(
Data file: TCD Day 35.CHR ()
Sample: RUN1
Comments: TYPE YOUR COMMENTS HERE
Temperature program:

Init temp	Hold	Ramp	Final temp
50.00	4.000	20.000	270.00

Events:

Time	Event
0.000	ZERO
0.000	D ON ()
0.200	G ON (ValveRotate)
7.000	G OFF (ValveRotate)

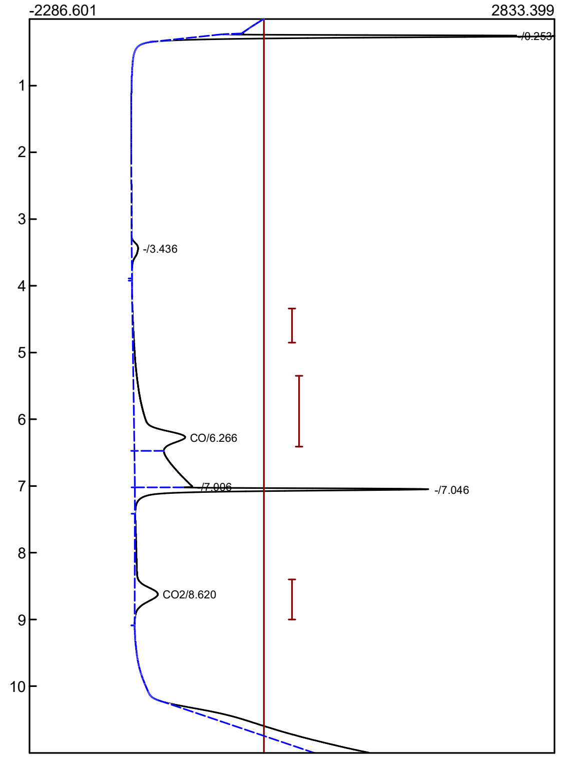

Component	Retention	Area	External	Units
-	0.253	8269.2390	0.0000	
-	3.436	870.0432	0.0000	
CO	6.266	12346.9760	0.0000	
-	7.006	13301.0690	0.0000	
-	7.046	8731.7305	0.0000	
CO2	8.620	4385.0413	0.0000	
-	11.786	87393.3334	0.0000	
-	12.206	1008.1385	0.0000	
-	12.246	2999.0468	0.0000	
-	12.400	932.3788	0.0000	
-	12.453	746.6624	0.0000	
-	12.506	774.3698	0.0000	
-	12.546	1115.5744	0.0000	
-	12.656	984.7073	0.0000	
-	12.743	426.5450	0.0000	
	12.793	208.1639	0.0000	
	12.846	181.6432	0.0000	
	12.890	190.6934	0.0000	
		144865.3559	0.0000	

Lab name: SRI Instruments
Client: Valued Customer
Client ID: N2024
Analysis date: 07/22/2021 11:19:04
Method: Syringe Injection
Description: TCD
Column: RESTEK 15METER MXT-1
Carrier: HELIUM AT 5 PSI
Integration: Peak sens=95.0 Base sens=60.0 Min area= 10.00 Standard= 1.000
Data file: TCD Day 35.CHR ()
Sample: RUN1
Comments: TYPE YOUR COMMENTS HERE

Temperature program:

Init temp	Hold	Ramp	Final temp

Events:

Time	Event
0.000	ZERO
1.000	INTEG IMMEDIATE
7.000	INTEG IMMEDIATE

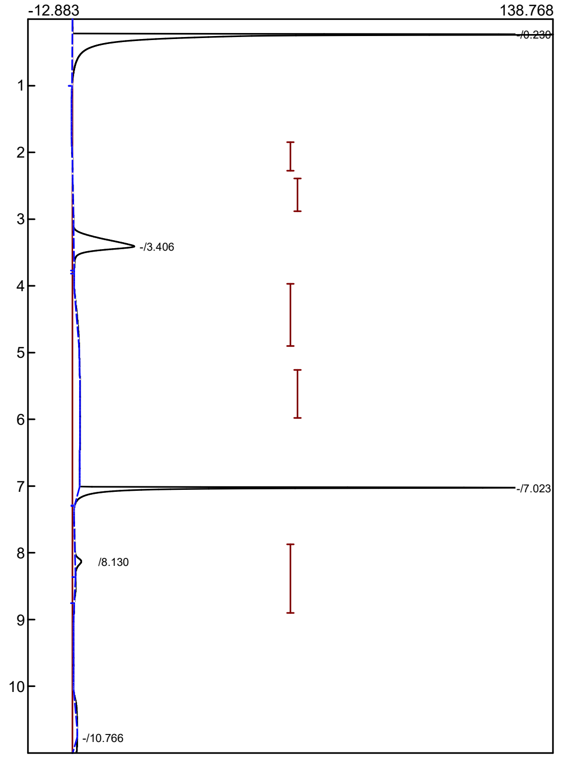

Retention	Area	External	Units
0.230	432.7396	0.0000	
3.406	185.2294	0.0000	
7.023	203.3418	0.0000	
8.130	15.3680	0.0000	
10.766	299.5242	0.0000	
	1136.2030	0.0000	

Fig. S5 GC-TCD spectra of the HCOOH decomposition. Reaction conditions: $\mathbf{B_1}$ (1 equiv.), **X*** (0.5 g), HCOOH (3 mL), Me-THF (3 mL), 110-130 °C and 16 h.

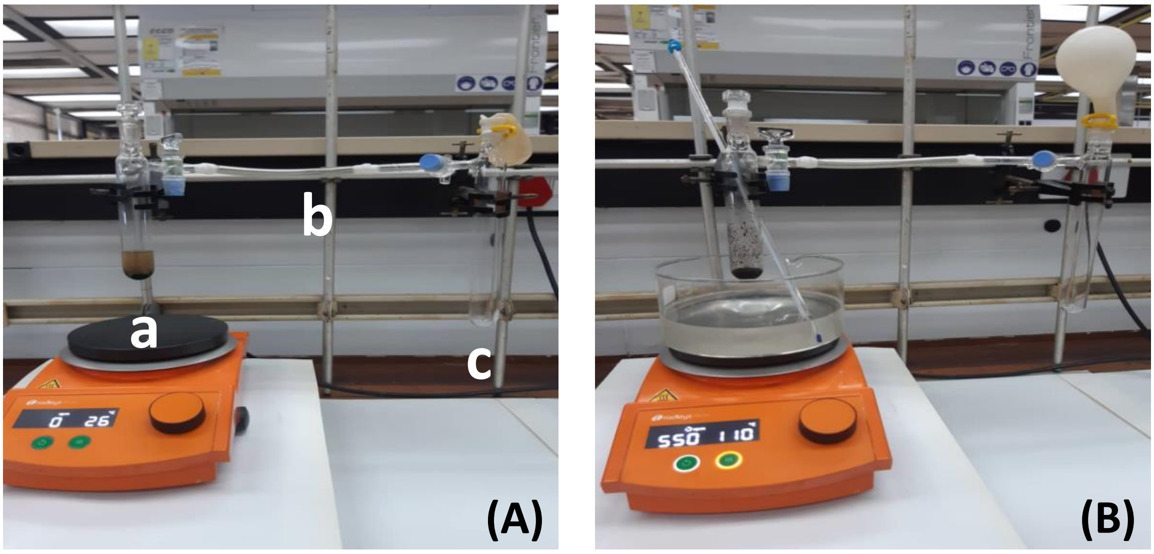

Fig. S6 3% Pd/Co_3O_4-Li catalyzed carbonylation reaction with formic acid in an open system experiment to show the formation of CO. Reaction conditions: Room temperature with all reactants in place **(A)**, and 110-130 °C, 12-16 h **(B)**. The inflated balloon in **(B)** shows the evidence of the CO release.

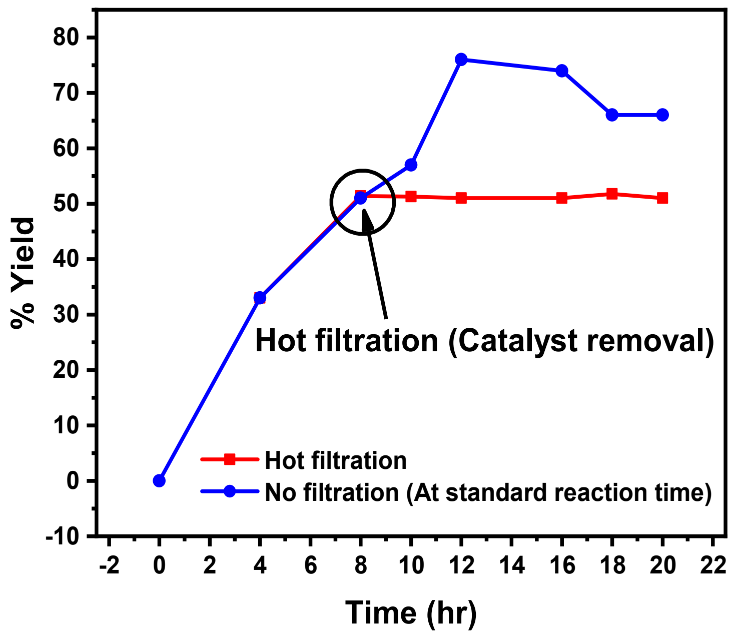

Fig. S7 Hot filtration test for the 3% Pd/Co_3O_4-Li catalyst.

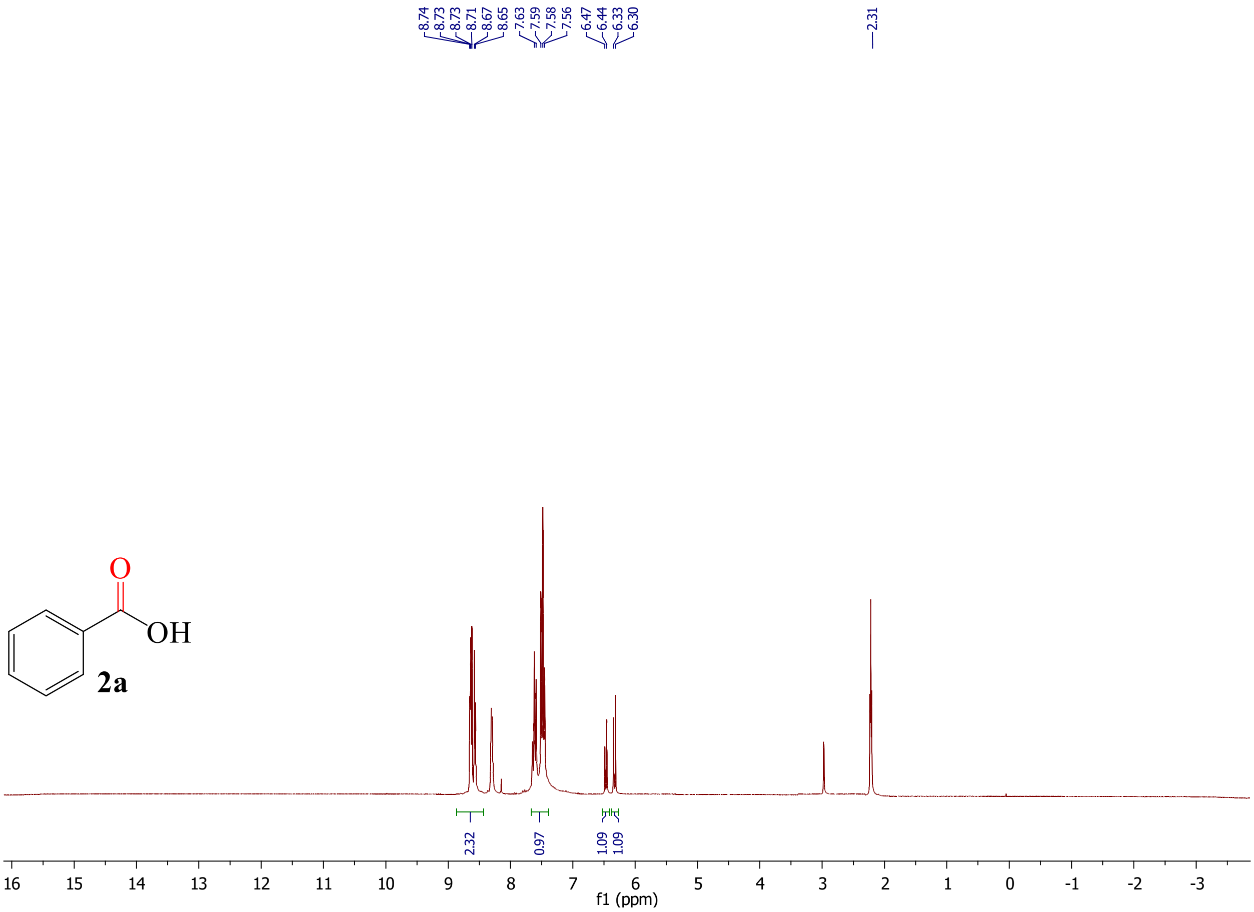
8.74
8.73
8.73
8.71
8.67
8.65
7.63
7.59
7.58
7.56
6.47
6.44
6.33
6.30
2.31
O
OH
2a
2.32
0.97
1.09
1.09
16 15 14 13 12 11 10 9 8 7 6 5 4 3 2 1 0 -1 -2 -3
f1 (ppm)

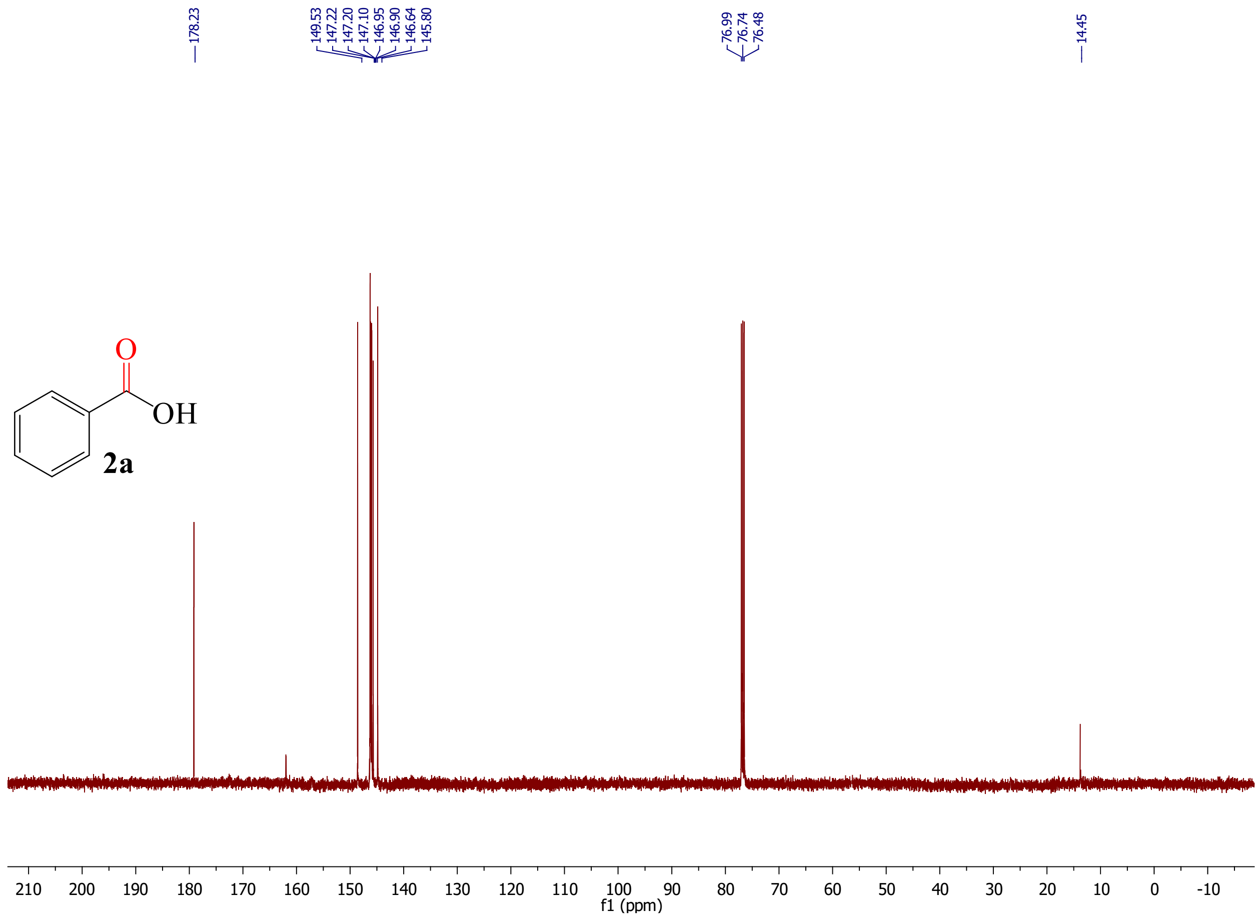
178.23
149.53
147.22
147.20
147.10
146.95
146.90
146.64
145.80
76.99
76.74
76.48
14.45
OH
O
2a
210 200 190 180 170 160 150 140 130 120 110 100 90 80 70 60 50 40 30 20 10 0 -10
f1 (ppm)

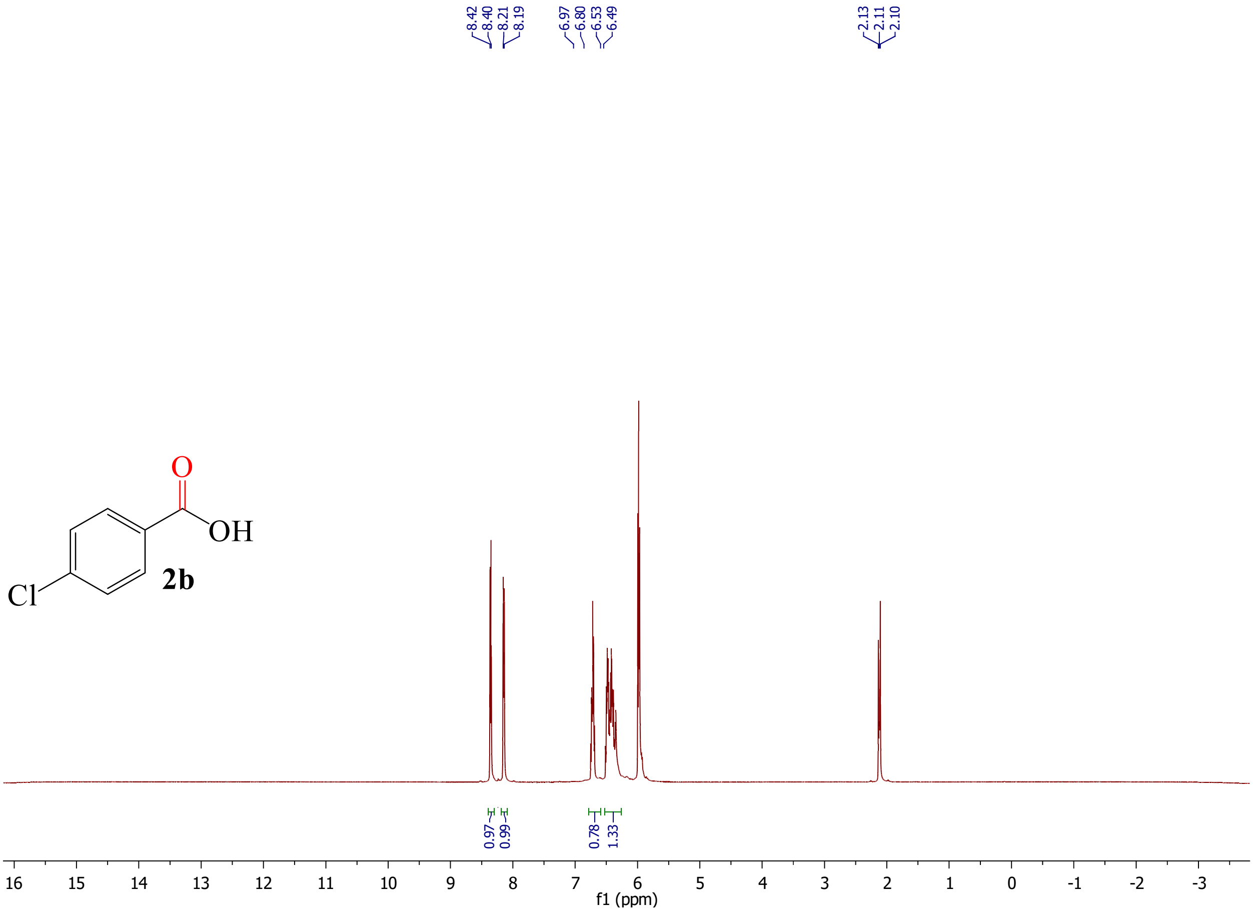
2b
8.42
8.40
8.21
8.19
6.97
6.80
6.53
6.49
2.13
2.11
2.10
0.97
0.99
0.78
1.33
16 15 14 13 12 11 10 9 8 7 6 5 4 3 2 1 0 -1 -2 -3
f1 (ppm)

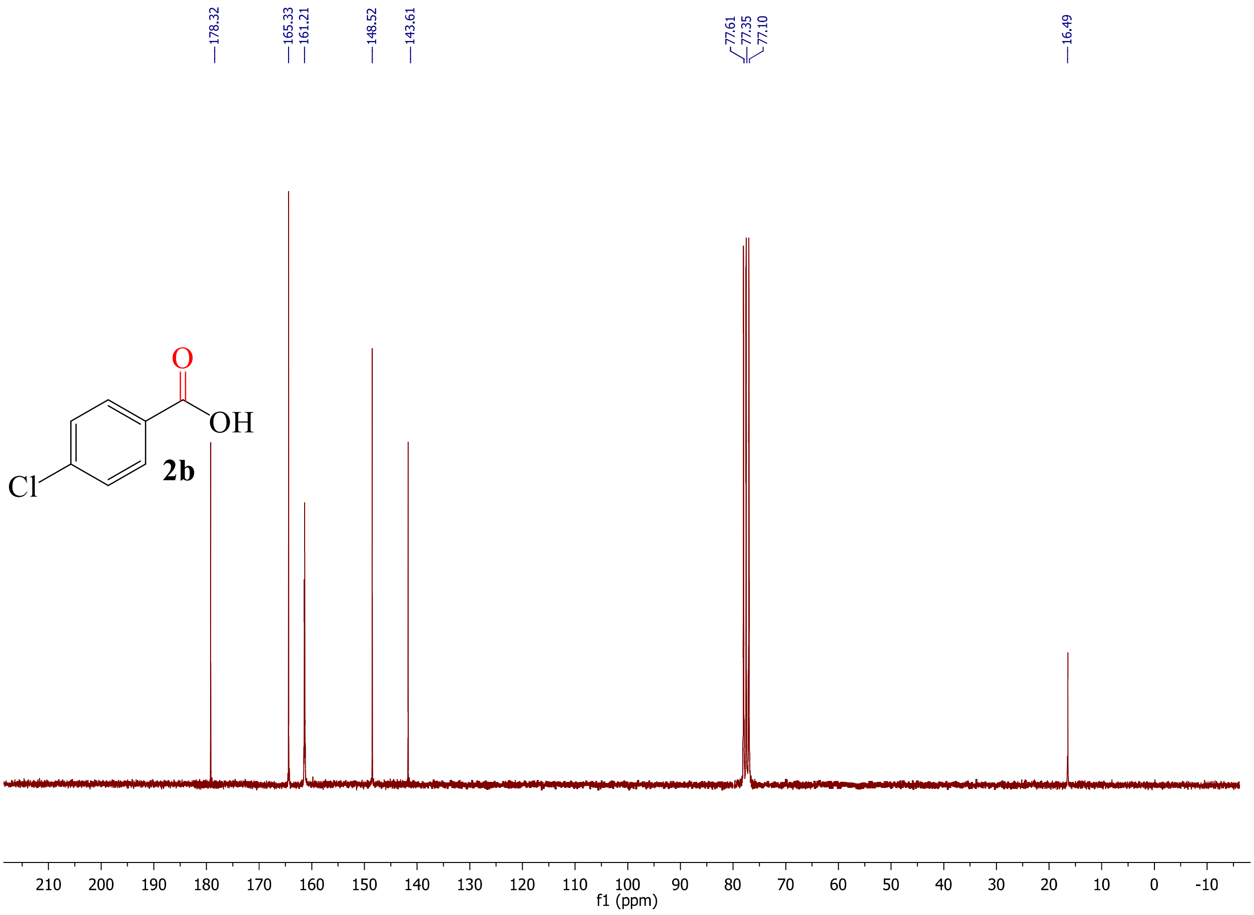
178.32
165.33
161.21
148.52
143.61
77.61
77.35
77.10
16.49
O
OH
Cl
2b
210 200 190 180 170 160 150 140 130 120 110 100 90 80 70 60 50 40 30 20 10 0 -10
f1 (ppm)

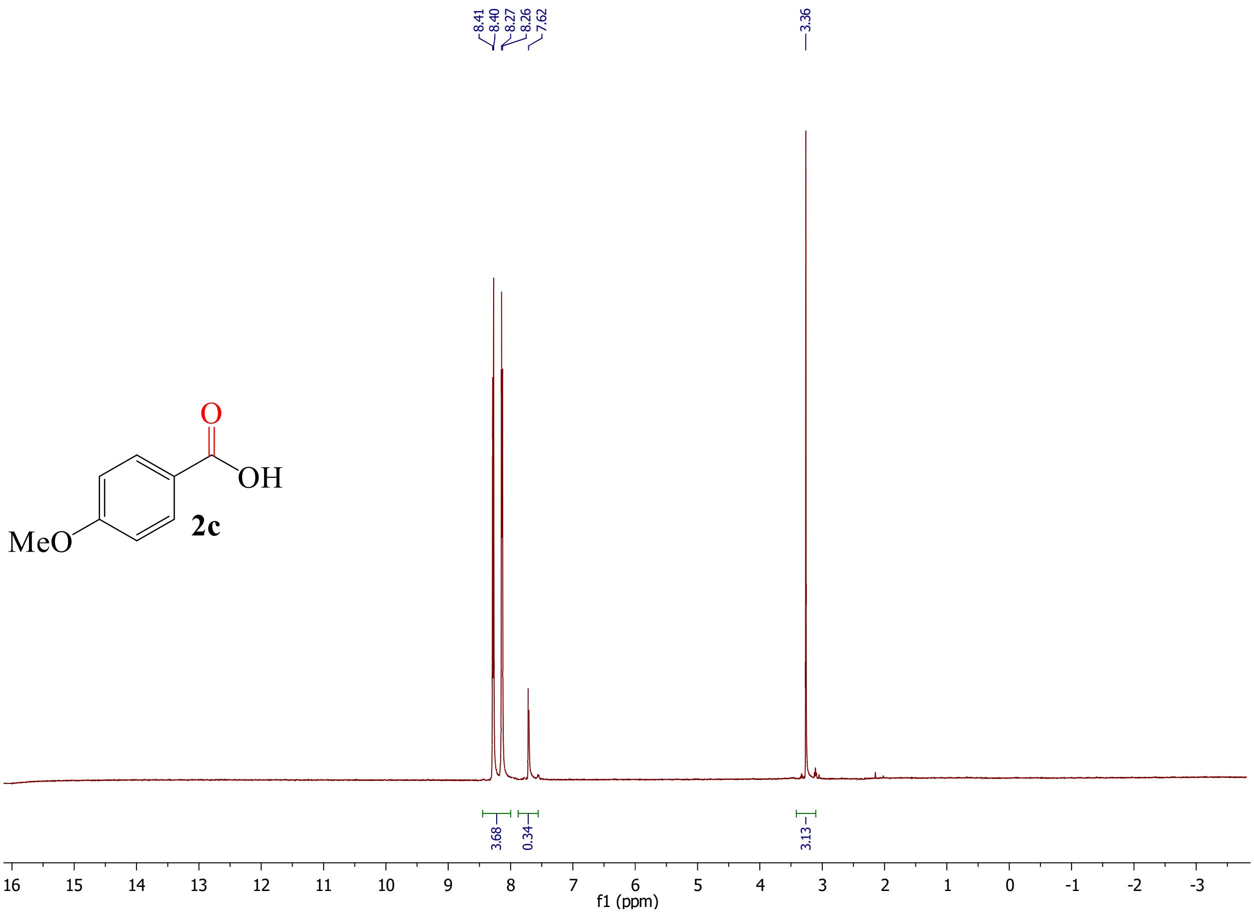
8.41
8.40
8.27
8.26
7.62
3.36
O
OH
MeO
2c
3.68
0.34
3.13
16 15 14 13 12 11 10 9 8 7 6 5 4 3 2 1 0 -1 -2 -3
f1 (ppm)

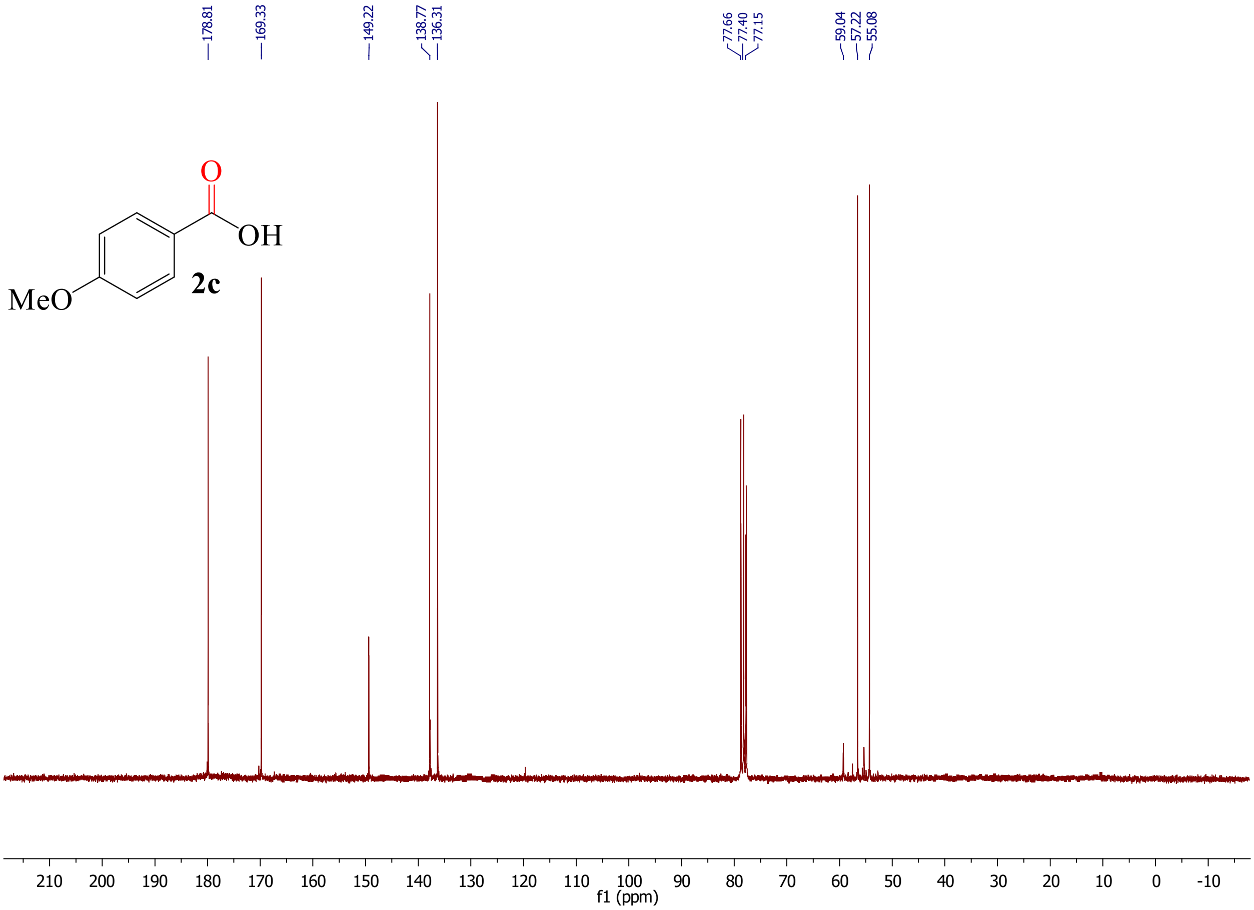
178.81
169.33
149.22
138.77
136.31
77.66
77.40
77.15
59.04
57.22
55.08
MeO
O
OH
2c
210 200 190 180 170 160 150 140 130 120 110 100 90 80 70 60 50 40 30 20 10 0 -10
f1 (ppm)

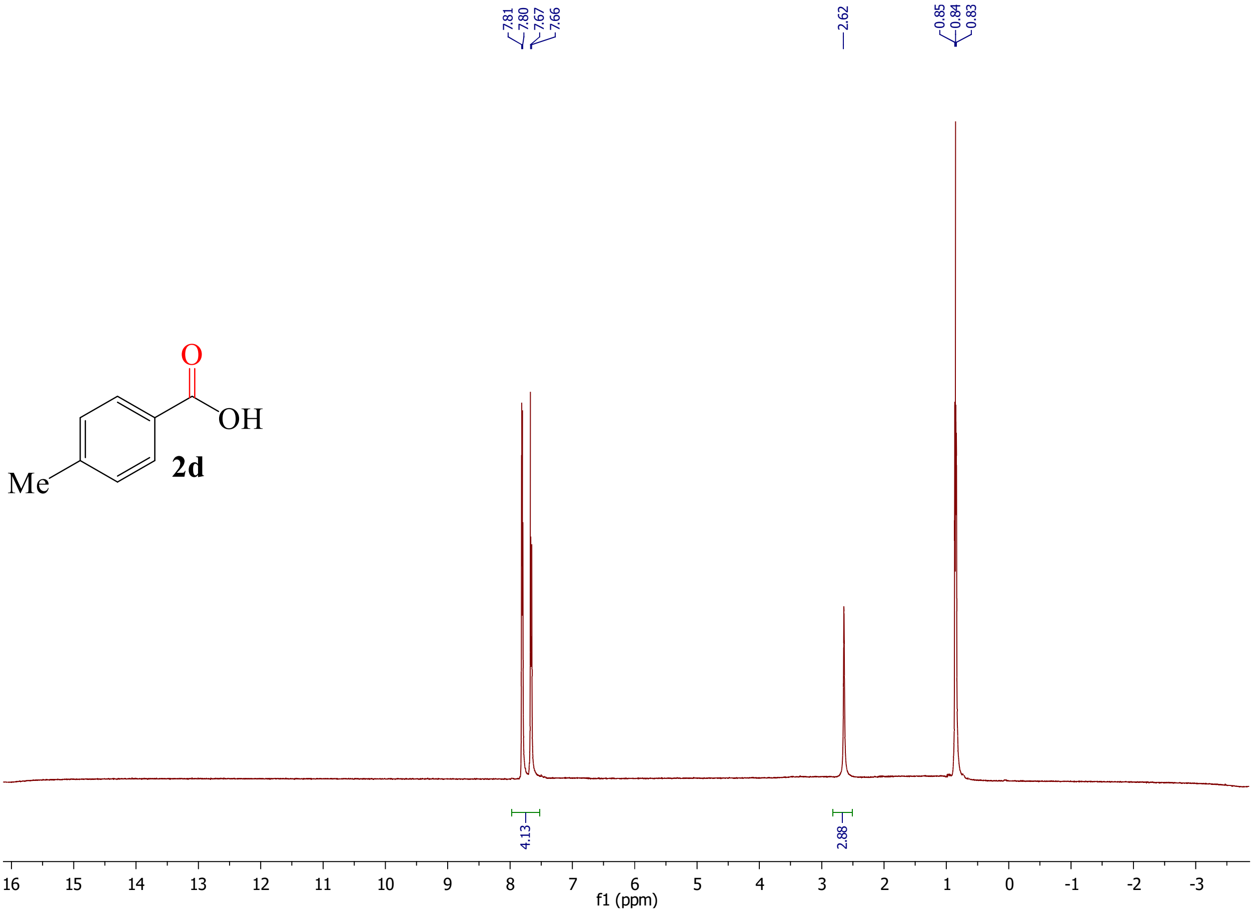
7.81
7.80
7.67
7.66
2.62
0.85
0.84
0.83
O
OH
Me
2d
4.13
2.88
16 15 14 13 12 11 10 9 8 7 6 5 4 3 2 1 0 -1 -2 -3
f1 (ppm)

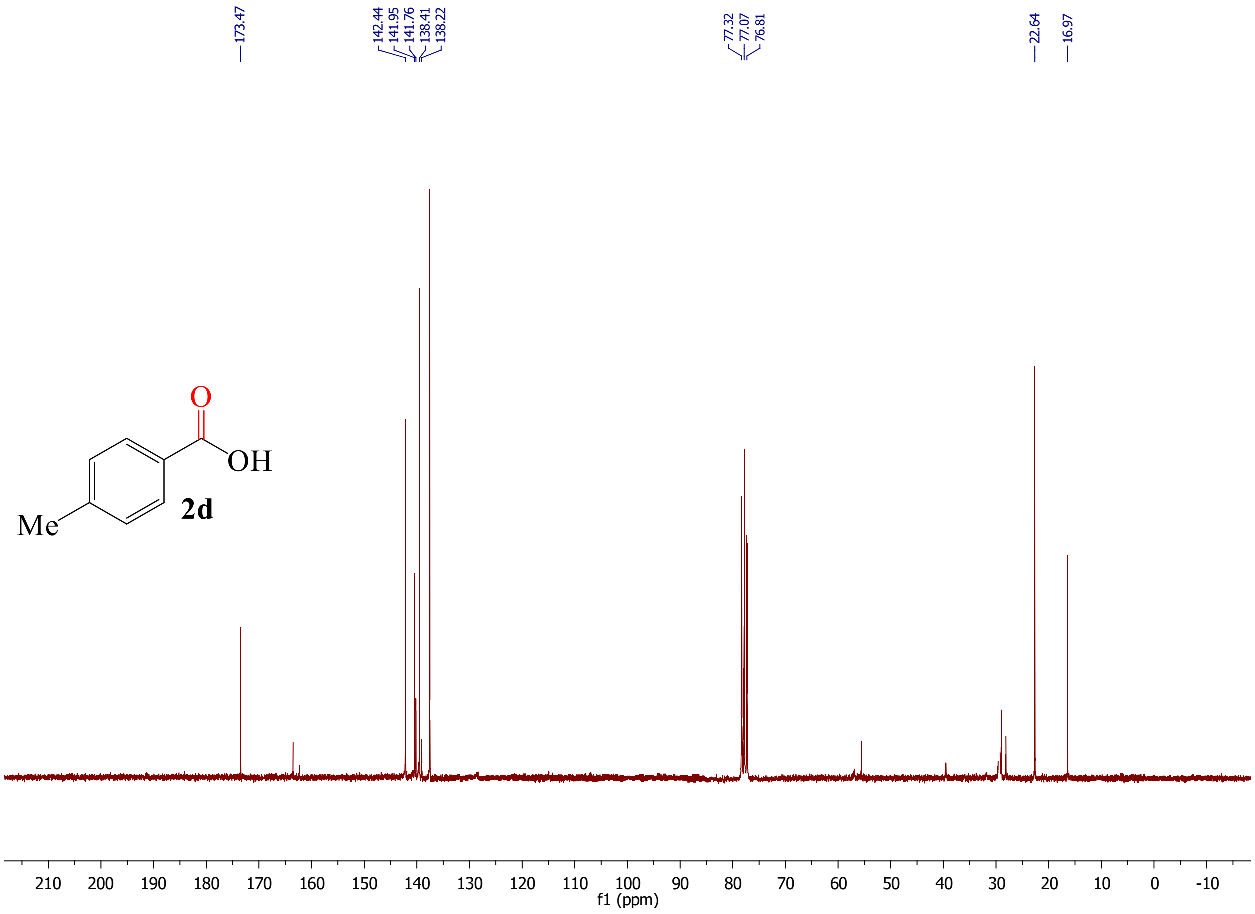
173.47
142.44
141.95
141.76
138.41
138.22
77.32
77.07
76.81
22.64
16.97
O
OH
Me
2d
210 200 190 180 170 160 150 140 130 120 110 100 90 80 70 60 50 40 30 20 10 0 -10
f1 (ppm)

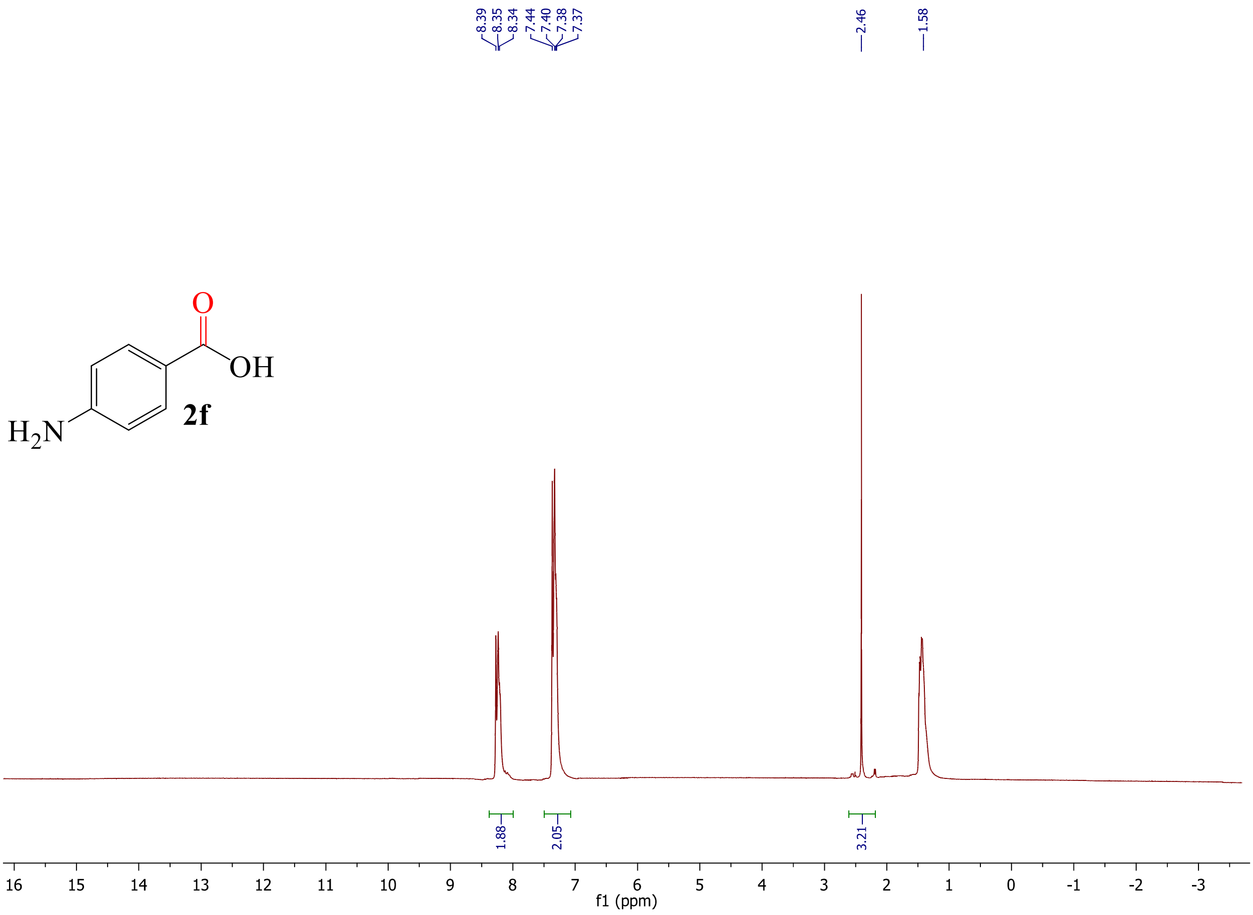

8.39
8.35
8.34
7.44
7.40
7.38
7.37
2.46
1.58
O
OH
H_2N
2f
1.88
2.05
3.21
16 15 14 13 12 11 10 9 8 7 6 5 4 3 2 1 0 -1 -2 -3
f1 (ppm)

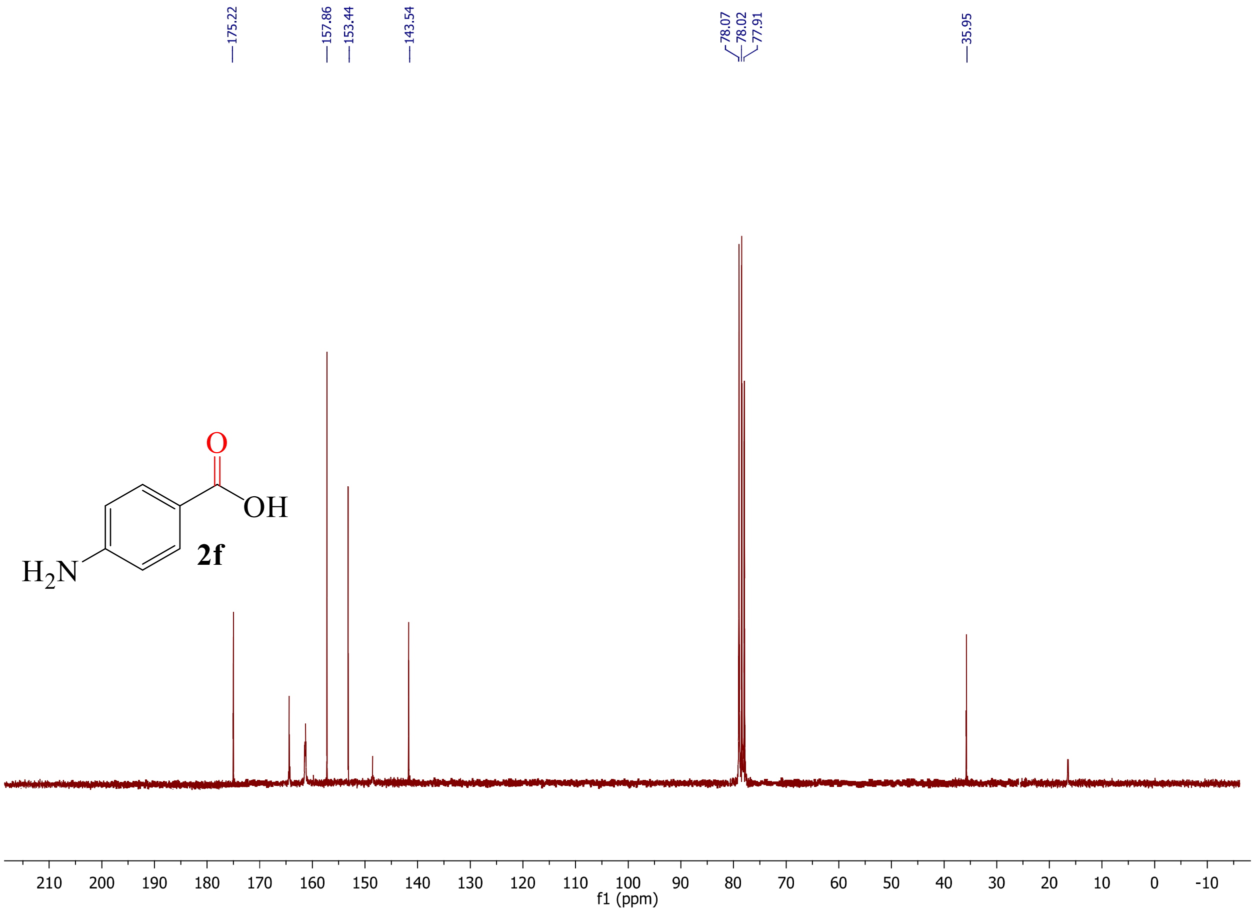
175.22
157.86
153.44
143.54
78.07
78.02
77.91
35.95
OH
O
H2N
2f
210
200
190
180
170
160
150
140
130
120
110
100
90
80
70
60
50
40
30
20
10
0
-10
f1 (ppm)

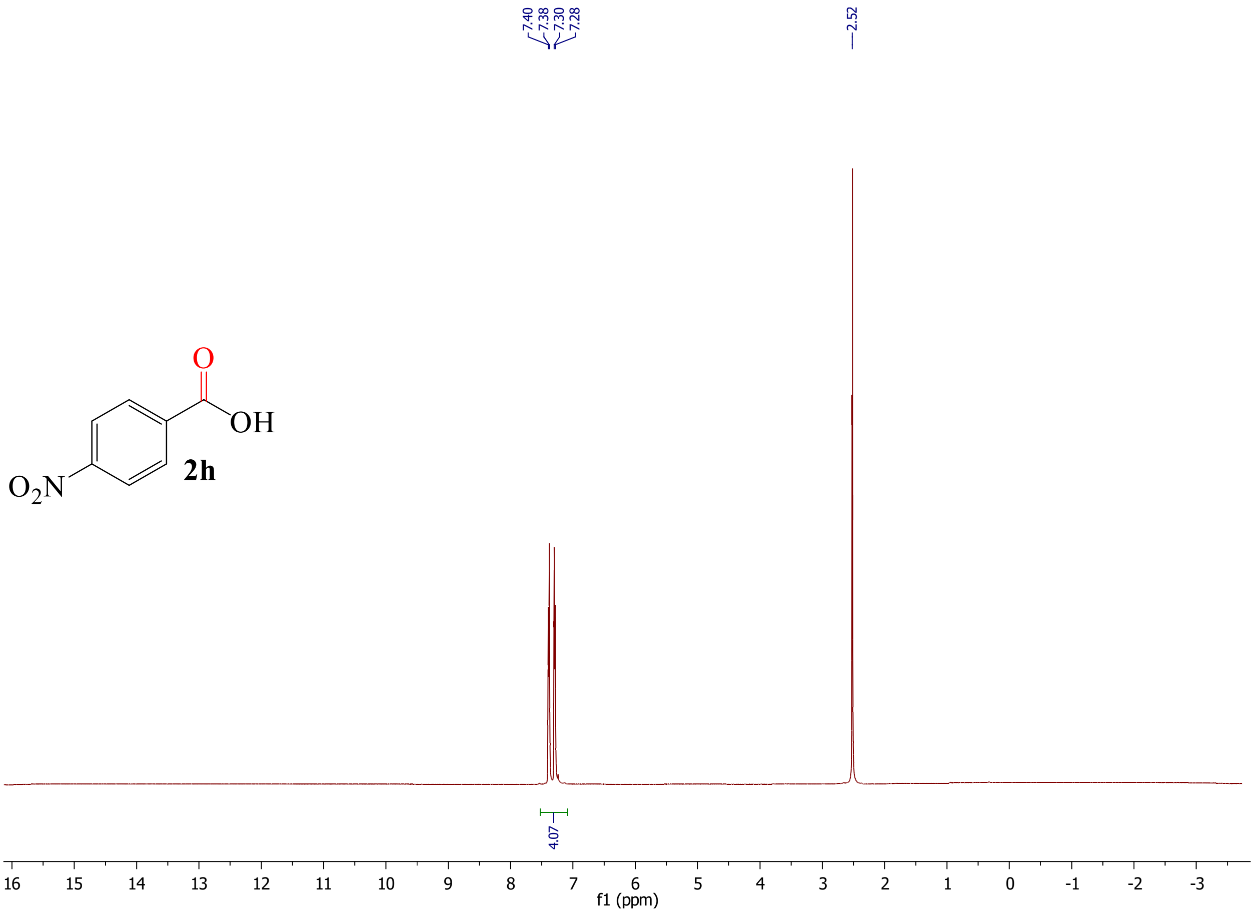
7.40
7.38
7.30
7.28
2.52
O
OH
O_2N
2h
4.07
16 15 14 13 12 11 10 9 8 7 6 5 4 3 2 1 0 -1 -2 -3
f1 (ppm)

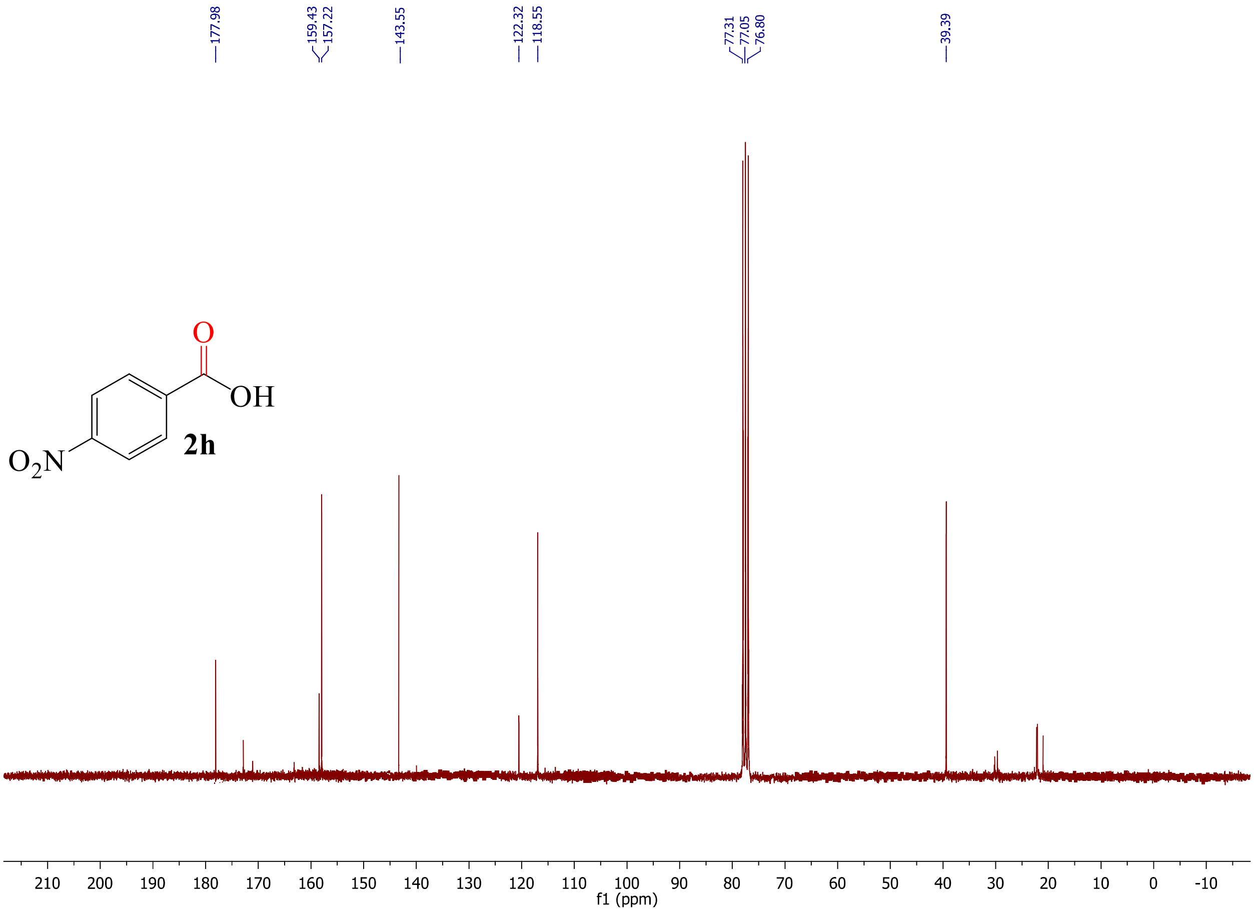
177.98
159.43
157.22
143.55
122.32
118.55
77.31
77.05
76.80
39.39
O
OH
O_2N
2h
210 200 190 180 170 160 150 140 130 120 110 100 90 80 70 60 50 40 30 20 10 0 -10
f1 (ppm)

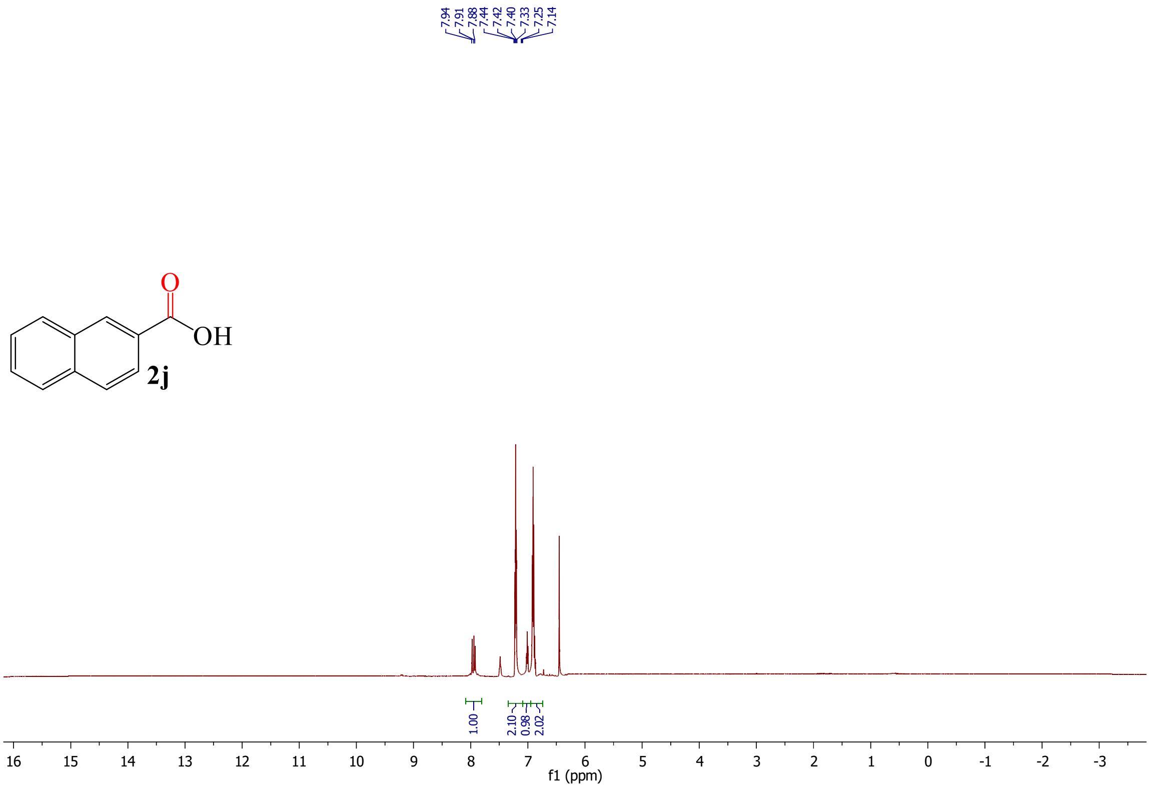
7.94
7.91
7.88
7.44
7.42
7.40
7.33
7.25
7.14
O
OH
2j
1.00
2.10
0.98
2.02
16 15 14 13 12 11 10 9 8 7 6 5 4 3 2 1 0 -1 -2 -3
f1 (ppm)

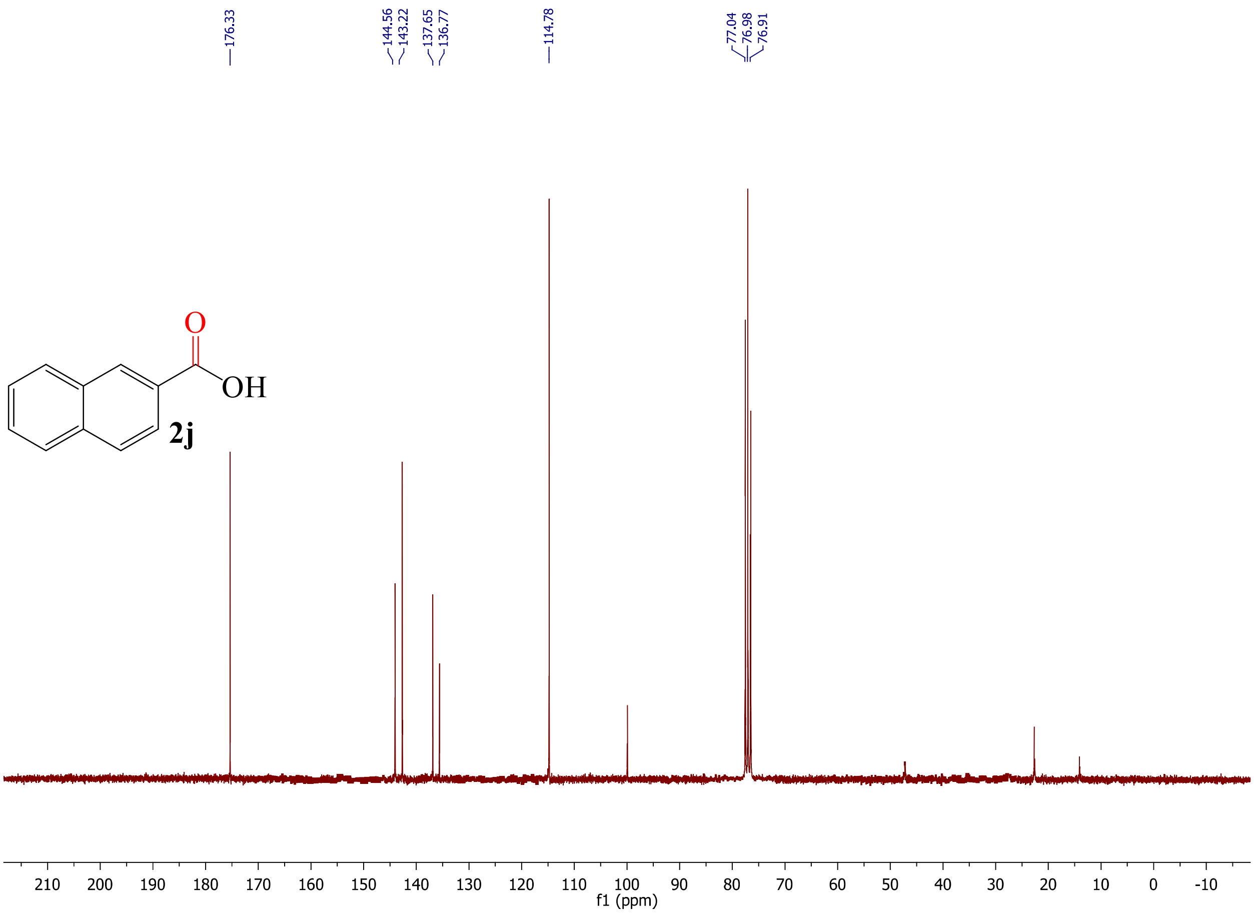
176.33
144.56
143.22
137.65
136.77
114.78
77.04
76.98
76.91
O
OH
2j
210 200 190 180 170 160 150 140 130 120 110 100 90 80 70 60 50 40 30 20 10 0 -10
f1 (ppm)

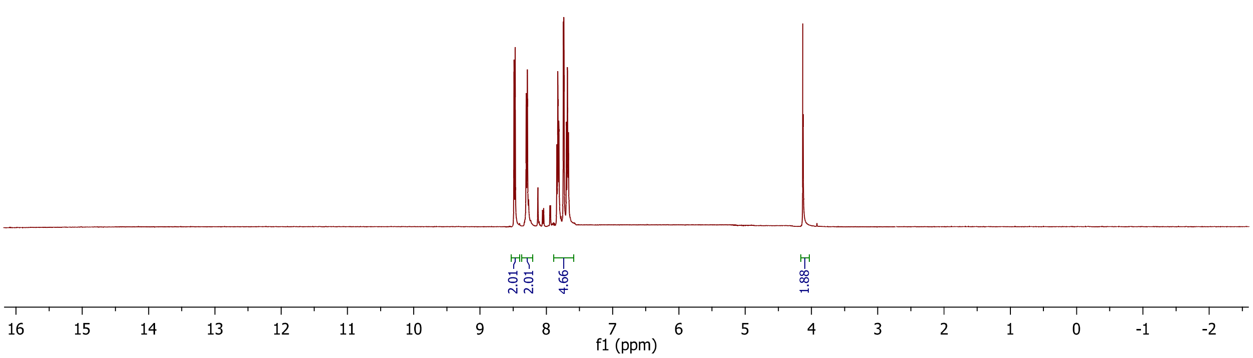
8.49
8.47
8.30
8.29
8.27
7.84
7.83
7.81
7.74
7.73
7.70
7.68
7.67
4.18
2.01
2.01
4.66
1.88
16 15 14 13 12 11 10 9 8 7 6 5 4 3 2 1 0 -1 -2
f1 (ppm)

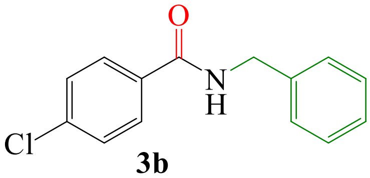
O
N
H
Cl

3b

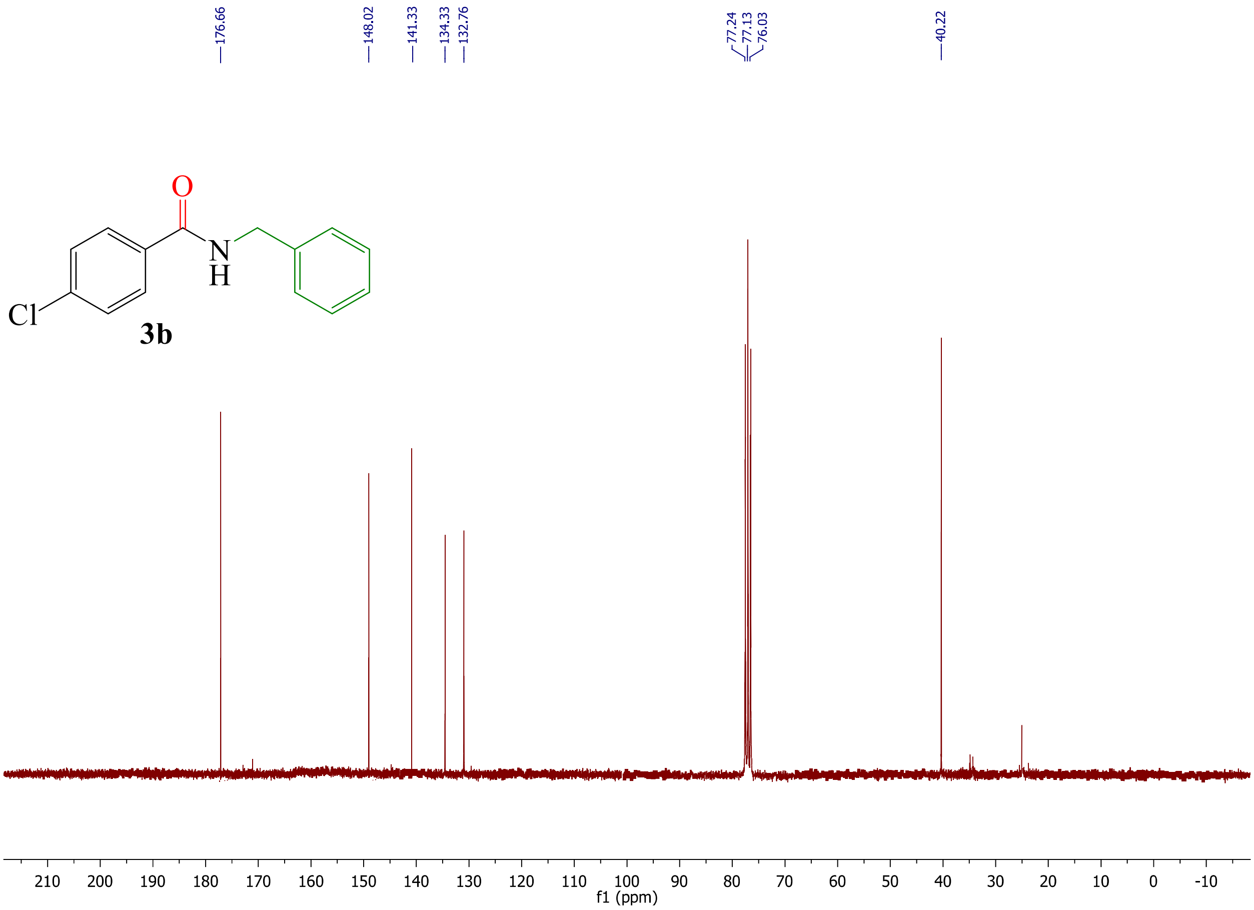
176.66
148.02
141.33
134.33
132.76
77.24
77.13
76.03
40.22
O
N
H
Cl
3b
210 200 190 180 170 160 150 140 130 120 110 100 90 80 70 60 50 40 30 20 10 0 -10
f1 (ppm)

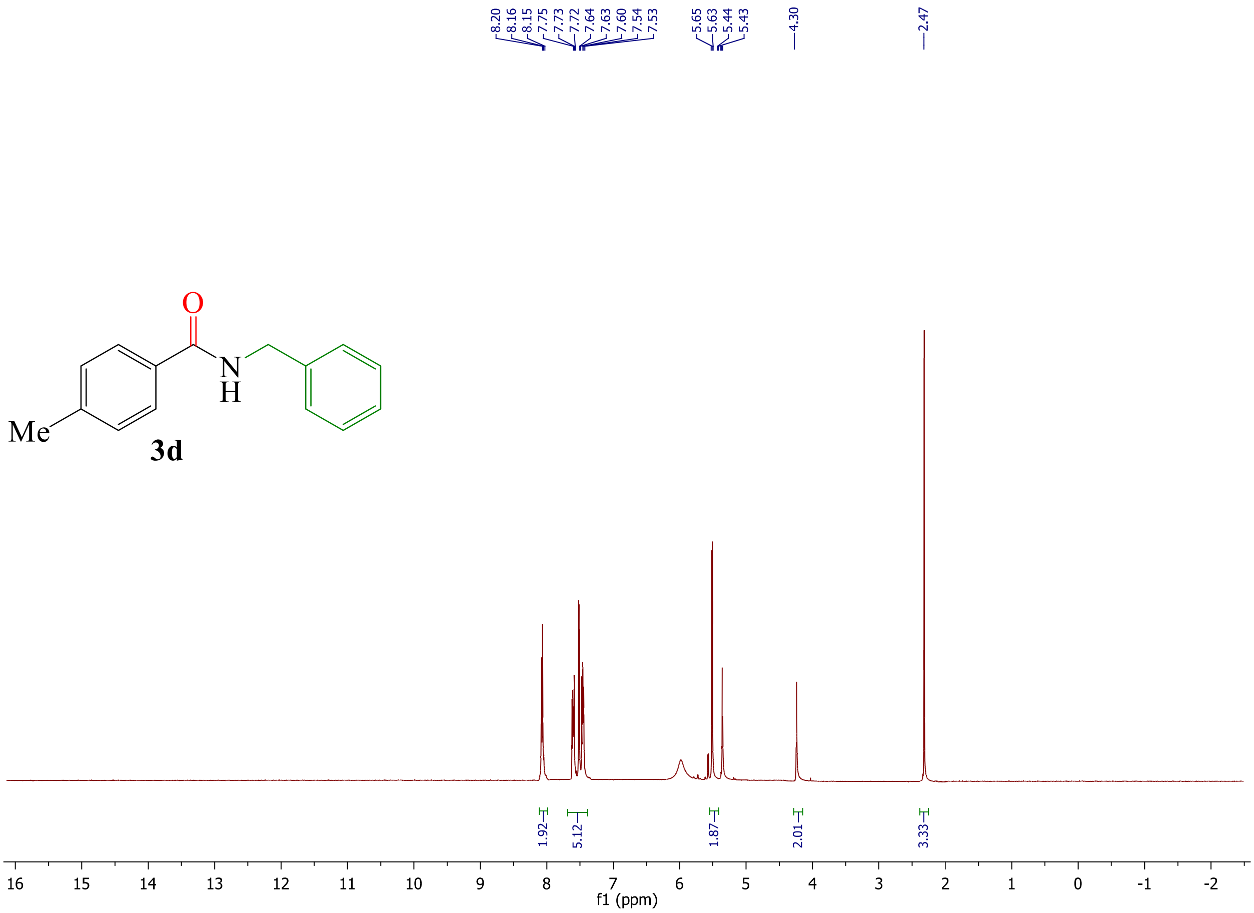
8.20
8.16
8.15
7.75
7.73
7.72
7.64
7.63
7.60
7.54
7.53
5.65
5.63
5.44
5.43
4.30
2.47
Me
N
H
O
3d
1.92
5.12
1.87
2.01
3.33
f1 (ppm)

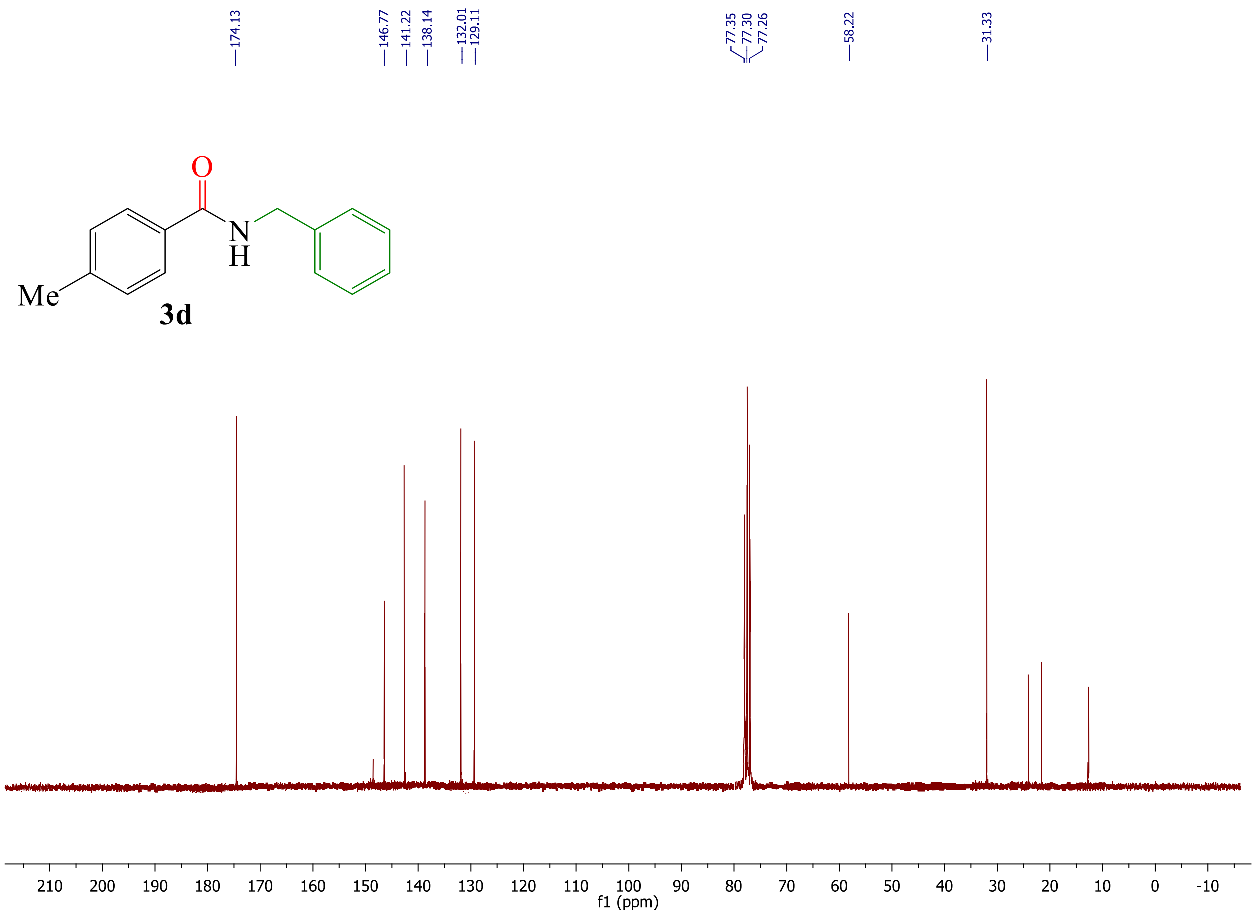
174.13
146.77
141.22
138.14
132.01
129.11
77.35
77.30
77.26
58.22
31.33
O
N
H
Me
3d
f1 (ppm)

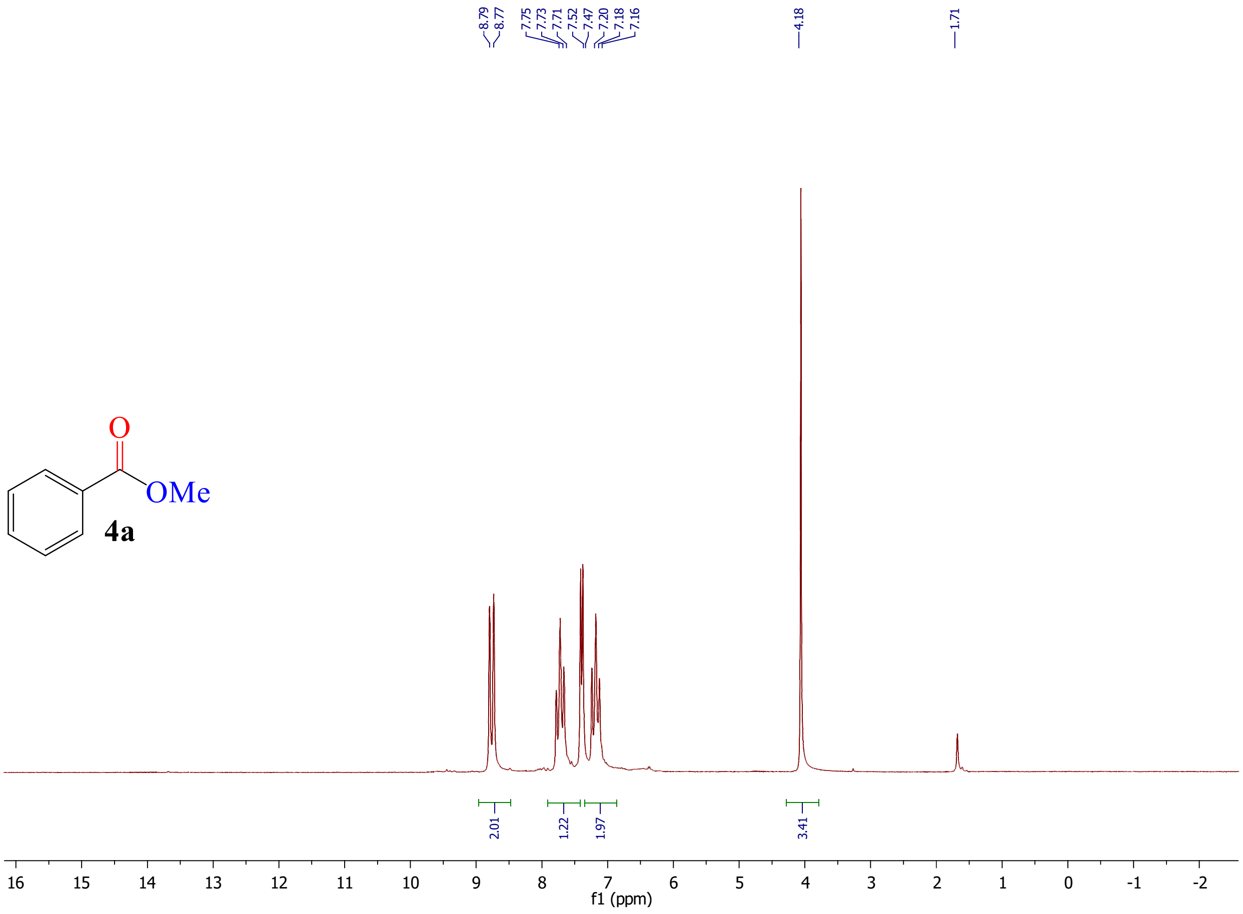

O
OMe
4a
8.79
8.77
7.75
7.73
7.71
7.52
7.47
7.20
7.18
7.16
4.18
1.71
2.01
1.22
1.97
3.41
16 15 14 13 12 11 10 9 8 7 6 5 4 3 2 1 0 -1 -2
f1 (ppm)

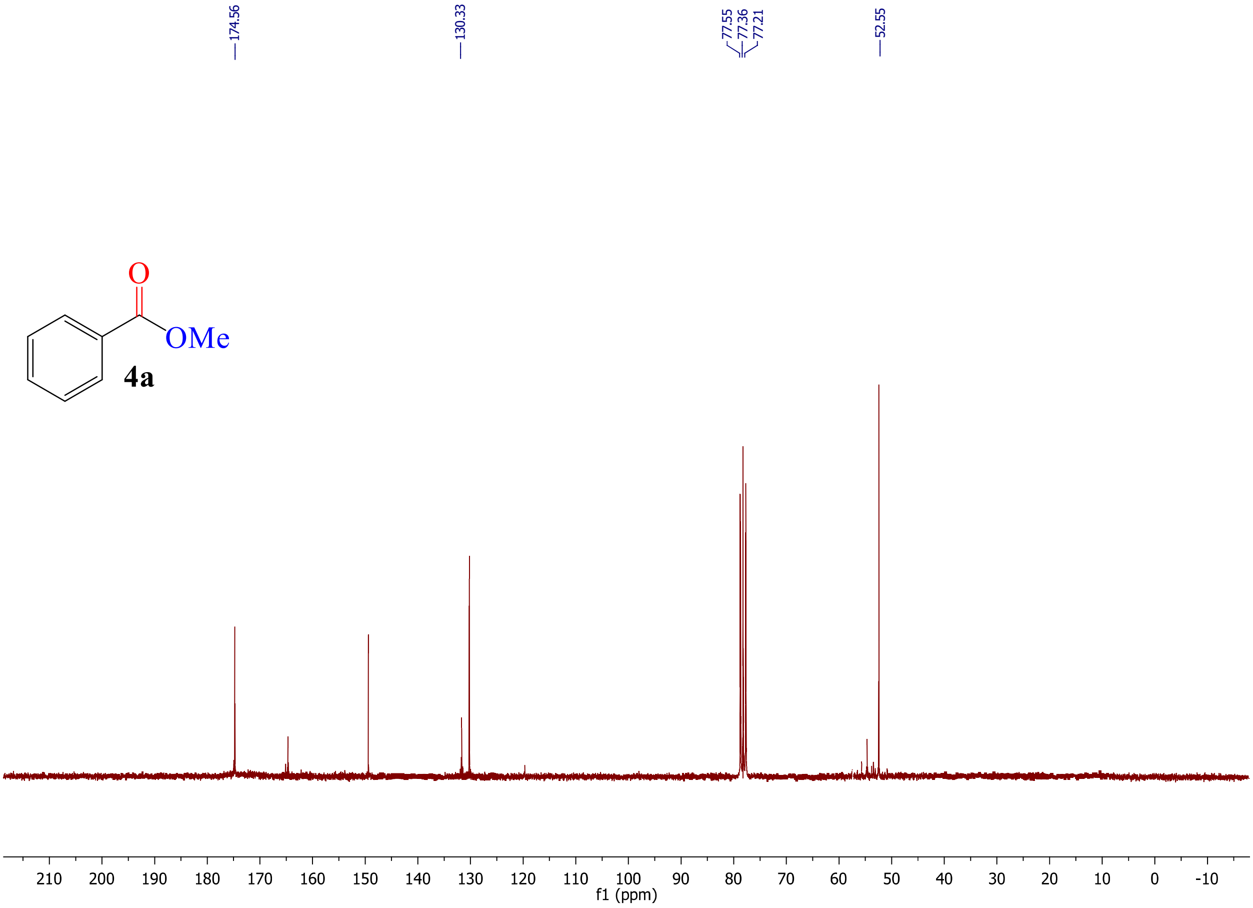
174.56
130.33
77.55
77.36
77.21
52.55
O
OMe
4a
210 200 190 180 170 160 150 140 130 120 110 100 90 80 70 60 50 40 30 20 10 0 -10
f1 (ppm)

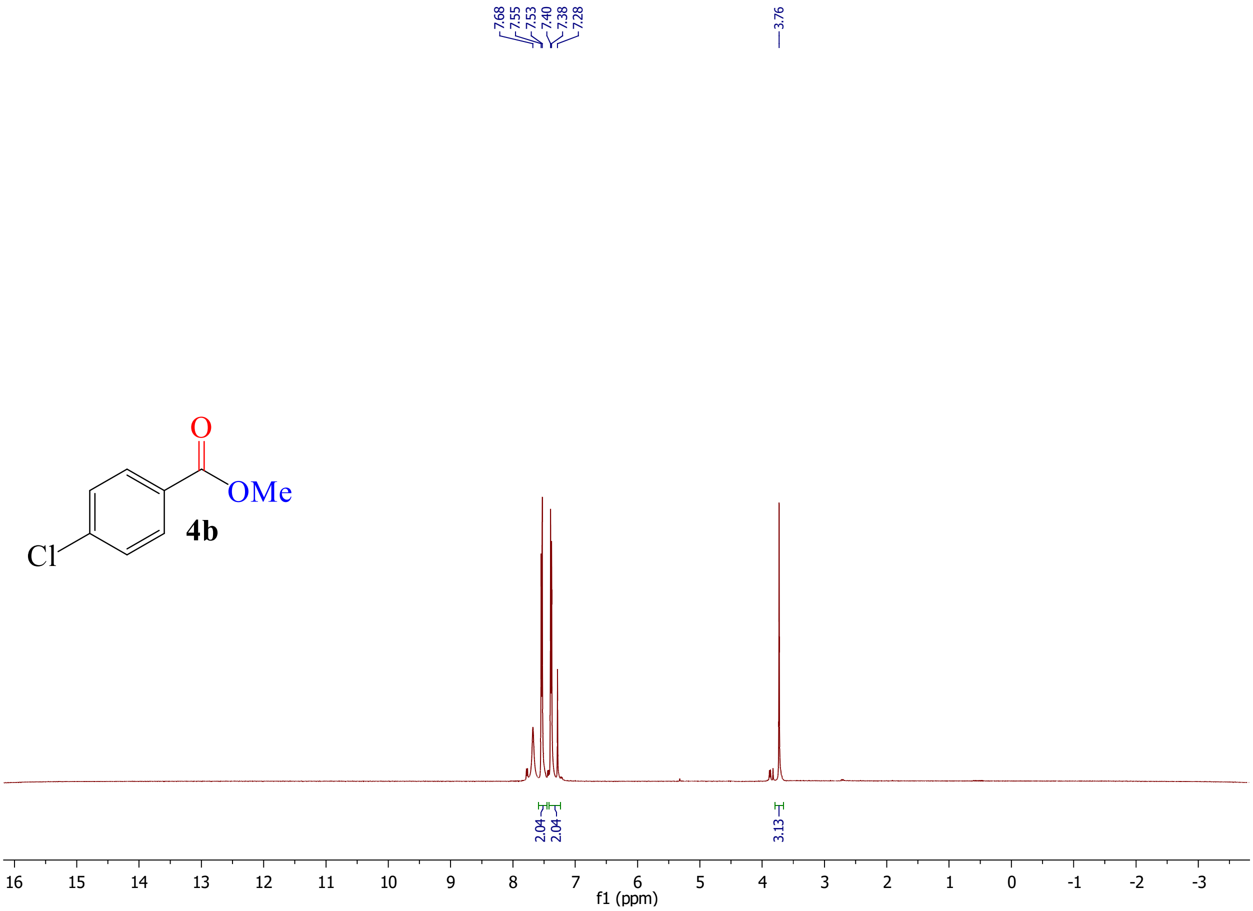

7.68
7.55
7.53
7.40
7.38
7.28
3.76
O
OMe
Cl
4b
2.04
2.04
3.13
16 15 14 13 12 11 10 9 8 7 6 5 4 3 2 1 0 -1 -2 -3
f1 (ppm)

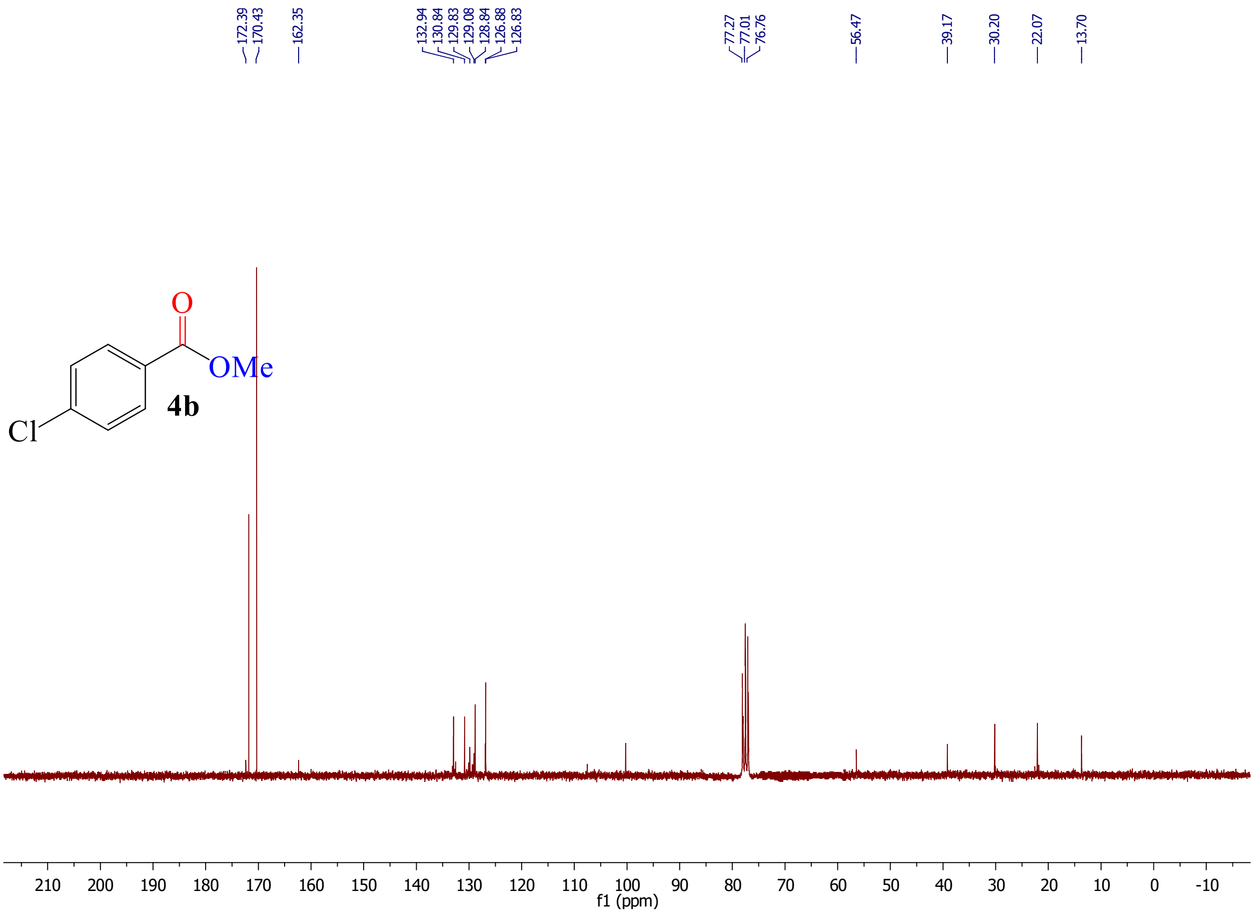
172.39
170.43
162.35
132.94
130.84
129.83
129.08
128.84
126.88
126.83
77.27
77.01
76.76
56.47
39.17
30.20
22.07
13.70
O
OMe
Cl
4b
210 200 190 180 170 160 150 140 130 120 110 100 90 80 70 60 50 40 30 20 10 0 -10
f1 (ppm)
